# A Systematic Review and Meta‐Analysis of Biological Sex Differences in Sleep Spindles and Slow Wave Activity in Adults With and Without Insomnia

**DOI:** 10.1111/jsr.70388

**Published:** 2026-06-22

**Authors:** Nyissa A. Walsh, Emma‐Maria Phillips, Arsenio Páez, Nathan E. Cross, Thien Thanh Dang‐Vu, Aurore A. Perrault

**Affiliations:** ^1^ Sleep, Cognition, & Neuroimaging Lab, Department of Health, Kinesiology & Applied Physiology & Centre for Studies in Behavioral Neurobiology Concordia University Montreal Quebec Canada; ^2^ Department of Psychology Concordia University Montreal Quebec Canada; ^3^ Centre de Recherche de l'Institut Universitaire de Gériatrie de Montréal Montreal Quebec Canada; ^4^ Department of Neurosciences, Faculty of Medicine Université de Montréal Montréal Quebec Canada; ^5^ Nuffield Department for Primary Care Health Sciences University of Oxford Oxford UK; ^6^ Brain and Mind Center, School of Psychology University of Sydney Sydney Australia; ^7^ Woolcock Institute of Medical Research Macquarie University Sydney Australia

**Keywords:** EEG, insomnia disorder, sex, sigma, sleep, slow oscillation, slow wave activity, spindle, systematic review

## Abstract

Mounting evidence shows sex‐based differences in sleep experiences and outcomes, including the prevalence of insomnia disorder. However, the impact of biological sex on brain oscillations during sleep remains poorly understood, especially in the context of insomnia disorder. This is a notable gap, given that neurophysiological aspects of sleep are associated with brain health and overall sleep quality. We systematically reviewed and meta‐analysed data from studies reporting spindle and slow wave activity in adults with and without insomnia disorder. We conducted systematic searches in PubMed, Embase, Scopus, and PsycInfo. Risk of bias was evaluated using the Newcastle Ottawa and the PEDro scales. Forty‐three studies met our inclusion criteria, with thirteen studies of normal sleepers (*N* = 668) reporting sufficient data for random‐effects meta‐analyses. Compared with males, female normal sleepers had higher spindle density, sigma and delta power. Most studies recruited individuals with primary insomnia, and data pooling for insomnia and mixed groups was not possible due to insufficient statistical reporting. Moreover, group‐by‐sex interactions were limited, inconsistent, and varied across studies and sample characteristics. Further research is needed to explore sex‐specific differences in sleep microarchitecture and their role in normal sleep and the manifestation of insomnia disorder.

**Abbreviations**
CBTi: cognitive‐behavioural therapy for insomnia
EEG: electroencephalography
EMG: electromyography
EOG: electrooculography
*F*
: female
IN: insomnia
*M*
: male
MeSH: Medical Subject Headings
NOS: Newcastle‐Ottawa Scale
NREM: non‐rapid eye movement
NREM2: non‐rapid eye movement stage 2
NREM3: non‐rapid eye movement stage 3
NS: normal sleeper
OA: older adult
PRISMA: Preferred Reporting Items for Systematic Reviews and Meta‐Analyses
PSG: Polysomnography
SO: slow oscillation
SW: slow wave
SWA: slow wave activity
YA: young adult

## 1 Introduction

Chronic insomnia or insomnia disorder is the most prevalent sleep disorder among adults affecting approximately 10%–17% of the population worldwide (Morin and Jarrin 2022; Benjafield et al. 2024). It is defined as self‐reported difficulties falling and/or staying asleep (insomnia symptoms), occurring more than 3 nights per week, persisting for at least 3 months, despite adequate opportunity to sleep, and a negative impact on daytime functioning such as mood disturbances and cognitive performance difficulties (American Academy of Sleep Medicine 2014; American Psychiatric Association 2013). Insomnia disorder affects approximately 2.5% of children aged 4 to 6, increasing to about 10% of children and adolescents aged 8 to 16, and even further during adulthood, indicating that clinically significant sleep disturbances can emerge prior to reproductive hormonal changes and evolve across development (Falch‐Madsen et al. 2020; de Zambotti et al. 2018). However, contrasting with childhood prevalence, the prevalence of chronic insomnia is one and a half times higher in females compared to males after puberty, where females often report poorer sleep quality than males in the general population (Morin and Jarrin 2022; Pajėdienė et al. 2024). The incidence of insomnia disorder differs by sex and age, with middle and older‐aged females at the highest risk for developing insomnia, compared to younger females and males of all ages (Morin and Jarrin 2022; American Psychiatric Association 2013).

In females, chronic insomnia is frequently reported during major reproductive life events marked by significant hormonal and physical changes, such as puberty, pregnancy, and perimenopause (Benge et al. 2024). Indeed, prior to the first menses (menarche), there is no difference between young females and males in the likelihood of developing insomnia, likely due to less hormonal fluctuations between sexes (Johnson et al. 2006). However, the risk of developing insomnia increases almost threefold in females after puberty and the establishment of menstrual cycles, while the risk for males remains relatively stable (Johnson et al. 2006; Krishnan and Collop 2006). Similarly, during the menopausal transition (perimenopause), there is a large increase in the risk of developing insomnia symptoms, from 35% in pre‐menopausal females to 53% in post‐menopausal females (Krishnan and Collop 2006). Menopausal transition is associated with decreases in levels of both progesterone and oestrogen, which are female reproductive hormones that have, among other functions, neurological protective effects. They increase synaptogenesis in brain regions important for memory and directly affect the brain regions associated with sleep (Harrington et al. 2022; Pletzer et al. 2023; Taxier et al. 2020). Thus, from adolescence to late life, fluctuations in gonadal steroids (such as oestrogen and progesterone) may be a contributing factor to the risk of developing insomnia disorder in females by influencing sleep physiology.

While insomnia diagnosis is solely based on subjective complaints, polysomnographic (PSG) recordings can reveal objective markers of sleep disturbances (e.g., longer sleep latency, more frequent awakenings) in adults with chronic insomnia (Hertenstein et al. 2018). In normal sleepers, sex‐based differences can already be observed at the macro‐architecture level, as it has been shown that females tend to go to bed earlier, fall asleep quicker and spend more time in bed. Compared to males, females also sleep longer and spend less time awake (i.e., greater sleep efficiency [SE]; total sleep time/total time in bed X 100%) (Goel et al. 2005; Manber et al. 2008; Carrier et al. 2017). Those differences are apparent in young healthy adults, but also in middle‐aged and older adults. Although sleep duration decreases with age regardless of sex, males tend to exhibit a somewhat significant linear decrease in total sleep time across the lifespan. In contrast, the sleep duration of females stays fairly stable until around age 50, then declines around menopause. By age 70, males and females have similar sleep durations (Bixler et al. 2009). Interestingly, although females tend to objectively sleep better than their male age‐matched counterparts across the lifespan (e.g., longer NREM3 duration from midlife onward) (Baker et al. 2016; Taporoski et al. 2025), they often report poorer sleep quality in the general population (Zhang and Wing 2006; Mallampalli and Carter 2014). This suggests that sex‐based differences in insomnia may be influenced not only by variations in sleep architecture, but also by psychosocial factors such as stress reactivity, hyperarousal, mood disturbances, and caregiving responsibilities, which may uniquely affect females experience of sleep disturbance (Benge et al. 2024).

While differences in sleep architecture and psychosocial factors are informative clinically, the investigation of brain oscillations offers a deeper understanding of the brain mechanisms underlying sleep depth, quality and sleep‐dependent memory processes (Djonlagic et al. 2021; Marshall et al. 2020). For instance, slow wave activity (SWA), a marker of homeostatic sleep drive (i.e., the pressure to sleep that builds during wakefulness and dissipates with sleep) reflects restorative NREM sleep and is linked to synaptic plasticity and memory consolidation (Timofeev and Chauvette 2017). SWA is composed of slow waves (SW; also called delta waves) oscillating between 1 and 4 Hz as well as high‐voltage biphasic waves called slow oscillations (SO; < 1.25 Hz, > 75 μV) (Dang‐Vu et al. 2008; Massimini et al. 2004; Rechtschaffen and Kales 1968). It is important to study both SW and SO activity as they originate from distinct neural mechanisms (Dang‐Vu et al. 2008). Indeed, SOs typically emerge from the prefrontal cortex, while SW stem from the thalamus and various cortical regions (Kim et al. 2019). Some persons with insomnia disorder show reduced or suppressed SWA, possibly due to the inability to fully disengage from a waking brain state, which may contribute to the feeling of non‐restorative sleep (Perlis et al. 2009). Whether studied as a waveform (i.e., event detection of SO < 1.25 Hz and slow waves < 4 Hz) or generalized activity over a longer period event (i.e., spectral power in the SO or delta frequency bands), SWA has often been associated with multiple cognitive and physiological functions, including synaptic homeostasis, metabolite clearance, and hormonal modulatory action (Léger et al. 2018).

Another crucial brain oscillation important for neural plasticity and restorative sleep is sleep spindles (Dang‐Vu et al. 2010; Fernandez and Lüthi 2020). Oscillating within the sigma range (11–16 Hz) between 0.5 to 3 s, spindles are abundant during NREM2 and NREM3 (Fernandez and Lüthi 2020). Sleep spindles are often categorized based on spatiotemporal characteristics; fast spindles (~14–16 Hz) occur mainly in the central and parital cortex, whereas slow spindles (~11–13 Hz) are located more frontally. Both spindles are key for understanding insomnia because their density, amplitude, and synchrony support stable, restorative sleep and protect against stress‐related disruptions (Dang‐Vu et al. 2010; Blanco‐Duque et al. 2024; Normand et al. 2016). Higher spindle activity correlates with deeper sleep and better perceived rest (Normand et al. 2016), while lower spindle density—especially early in the night—predicts greater vulnerability to stress and environmental disturbances, both of which can worsen insomnia symptoms (Dang‐Vu et al. 2015). However, studies on spindle activity in individuals with insomnia disorder are inconsistent; some find fewer or weaker spindles, while others find no differences compared to good sleepers (Weiner and Dang‐Vu 2016). Despite these inconsistencies, spindles may still be useful for predicting who will respond well to cognitive‐behavioural therapy for insomnia (CBTi) (Dang‐Vu et al. 2017), because they are thought to regulate sleep stability by filtering external stimuli (Dang‐Vu et al. 2015).

Taken together, hormonally mediated electrophysiological changes may contribute to broader sleep disturbance or circadian dysregulation (Pajėdienė et al. 2024) but may be more directly implicated in insomnia by disrupting sleep stability and thalamocortical inhibitory processes (Dang‐Vu et al. 2010; Blanco‐Duque et al. 2024; Normand et al. 2016), thereby increasing arousability and sleep complaints. These biological factors likely interact with psychosocial processes (e.g., stress, mood, and behaviour) to further increase vulnerability to insomnia.

### 1.1 Evidence Gap

Given the high prevalence of insomnia in females, this study fills a critical gap in sleep research as the first systematic synthesis and meta‐analysis to investigate sex‐based differences in spindle and SWA extracted from EEG recordings across both adults with insomnia and normal sleeping populations. To address this critical evidence gap, we aimed to determine whether measures of spindle activity (including spindle event and sigma power) and SWA (including SO, SW, delta power) differ between biological sex (i.e., female, male) in adults with and without insomnia. While prior reviews have focused on macro‐sleep architecture or neural oscillation alterations in insomnia without stratifying by sex, our work is the first synthesis to integrate sex and neural oscillations in insomnia, to explain why females tend to report poorer sleep quality and higher insomnia risk, despite often having better objective sleep than males. Conducting a systematic review of the current evidence will help pinpoint priority areas for future research and enhance evidence‐based treatment of insomnia disorder.

## 2 Methods

The systematic review was conducted following the recommendations of the Cochrane Handbook for Systematic Reviews of Interventions (Higgins et al. 2021). Results are reported in accordance with the Preferred Reporting Items for Systematic Reviews and Meta‐Analyses (PRISMA) guidelines (Page et al. 2021). The protocol was registered on the NIHR‐PROSPERO prospective register of systematic reviews (https://www.crd.york.ac.uk/PROSPERO/view/CRD42022355969).

### 2.1 Inclusion Criteria

Population (Morgan et al. 2018): Human adults over 18 years old, with or without insomnia disorder. Papers including younger individuals were eligible if data extraction was possible for the target age group or if the mean age was 18 years old or greater.

Exposure: Females.

Comparison: Males.

Outcomes: Eligible papers had to report continuous data for spindle measures (i.e., spindle density, amplitude, frequency, peak frequency, count, duration, laterality, distribution, diffuseness, as well as absolute, relative, and normalized sigma power) and/or SWA measures (i.e., SO and SW density, SW amplitude, frequency, phase duration, slope, incidence, distribution, laterality, and diffuseness as well as absolute, relative, and normalized delta power and SO power) recorded during sleep studies.

Study design: Only peer‐reviewed, cross‐sectional, case–control, cohort, or randomized‐controlled trial (RCT) studies were eligible for the current review. Pharmacological/psychological intervention studies were included if they reported baseline (pre‐treatment) information about participants meeting our inclusion criteria. To be eligible for the meta‐analyses, papers included in the systematic review had to report sex‐specific data on brain oscillations in normal sleepers and/or persons with insomnia.

### 2.2 Exclusion Criteria

Meeting abstracts, posters, case reports, editorial, commentary, research theses, and narrative review papers were excluded. Papers not reporting sex‐based analyses of sleep spindle or SWA we deemed ineligible. Studies with participants under the age of 18 or those including only males or only females were excluded as cross‐study sex comparisons risk conflating biological differences with methodological artefacts (e.g., measurement differences in spindle or SWA). Given the effects of chronic diseases and associated treatments on sleep, we excluded studies on participants with pre‐defined major medical disorders (e.g., cancer, chronic pain; major neurological disorders such as– epilepsy, dementia, traumatic brain injury; major psychiatric disorders such as– schizophrenia, major depression, bipolar disorder) (American Psychiatric Association 2013). Studies assessing treatment effects on PSG/EEG outcomes were excluded, but baseline data were retained. Given both positive and negative effects of treatments/interventions on sleep, mood, and memory, including such cases may have confounded the results of this review (Fleming et al. 2014). Therefore, post‐treatment outcomes on participants undergoing interventions or treatments were not included. Similarly, we excluded studies with participants using known drugs that affect sleep physiology (e.g., antidepressant, hypnotic‐sedative medications). For example, benzodiazepines, commonly prescribed sleep‐inducing drugs, have been shown to alter sleep spindles during NREM (Barbaux et al. 2025; Plante et al. 2015). Sleep medications were also an exclusion criterion. Articles focused on sleep disorders (OSA, CRDs, parasomnias etc.) other than insomnia were excluded. Only papers containing broad spectrum EEG variables (e.g., delta and sigma power) and NREM events (i.e., SO, SW and spindle density, duration, amplitude, frequency, phase duration, slope, incidence, distribution laterality, and diffuseness) were included. Therefore, studies that only examined sleep architecture variables (e.g., total sleep time, duration of sleep stages) or extracted spectral activity within EEG frequency bands not of interest (e.g., alpha) were removed. Studies indicating “slow wave sleep” were initially included for further investigation to ensure they reported data for SWA either as an event‐ or spectral power‐based rather than NREM3 stage percentage, which is often reported as SWA within sleep literature. If only the percentage of time spent in NREM3 was reported, the study was excluded, as this metric does not directly reflect SWA. In total, 83 studies were excluded at this stage due to reporting EEG variables that did not meet inclusion criteria (e.g., beta power, theta power), of which 5 studies (6.0%) specifically used the term “SWA” to refer to NREM3 duration. For the meta‐analysis, if eligible studies did not report summary statistics (e.g., means, SDs), we attempted to contact authors to obtain the missing data. If data remained unavailable, studies were excluded from meta‐analysis but included in the systematic review.

### 2.3 Information Sources

Systematic searches were conducted on PubMed, Embase, Scopus and PsycInfo from inception to November 2025. To ensure literature saturation, we also undertook citation searching of the reference lists of 2 relevant reviews that were excluded from our systematic review—specifically, a meta‐analysis on sleep microarchitecture in insomnia (Baglioni et al. 2014) and a systematic review on sex‐based differences in the sleep–wake cycle (Carrier et al. 2017)—to identify any additional eligible studies not captured by our primary search.

### 2.4 Search Strategy

Searches were undertaken with Medical Subject Headings (MeSH) terms, and keywords related to brain oscillations in persons with and without insomnia (See Figure S1). Keywords for biological sex analyses were not included to avoid missing papers reporting sex‐based differences but not describing them in their methods. Papers written in any language were eligible without a publication date restriction. Studies published in languages other than English were translated using 2 independent translation tools to ensure accuracy. None of the non‐English articles met the inclusion criteria for the final systematic review or meta‐analysis.

### 2.5 Selection Process

After the removal of duplicate results using the Zotero Software (Version 6.0.37), the following selection process was applied:

First, search results were screened against our inclusion and exclusion criteria by title and abstract. Papers meeting those criteria, or papers for which we needed more information to make a determination, were then selected for full‐text screening. Authors were contacted to obtain additional information regarding the study's method to assess eligibility when necessary. The first stage of the selection process was conducted by 2 reviewers (N.A.W., E.M.P.). Agreement between study eligibility was based on a consensus between 3 authors (N.A.W., E.M.P., A.A.P.), and any disagreements were resolved by consensus from all 6 authors.

### 2.6 Data Extraction

Data extraction was undertaken and checked by the first reviewer (NAW) and second investigator (EMP). Using Zotero Software and customized Microsoft Excel sheets created following PRISMA guidelines. Study characteristics included the author name, publication year, study location, population demographics (e.g., frequency of biological sex, average age, age range, sample size), study design, the method used to measure brain oscillations (e.g., number of nights, sampling rate, electrodes used, epochs time length for scoring, stages used for scoring [NREM2, NREM3 or both]), the target variables of interest (e.g., spindle density, SO amplitude), and the main outcomes corresponding to the aims of the systematic review.

Given the frequent interchangeability of the terms sex and gender in the insomnia literature, we used the terms women, females, men, and males to categorize participants based on biological sex. Since none of the included studies examined the societal influences of gender on sleep, our analyses were grounded in biological sex rather than gender as a social construct.

Given heterogeneity in terms for brain oscillations found in the sleep literature (i.e., non‐systematic use of similar terms to define spindle, sigma, SO, delta and SWA), any outcomes (e.g., density, amplitude, peak frequency, duration) referring to an event detection were either categorized as spindles (detected within sigma range), slow waves (SW; detected within 0.25–4 Hz) or SO (detected below 1.25 Hz). Meanwhile, if power spectral analyses were used, they were categorized as sigma (~11–16 Hz), delta (~0.25–4 Hz), or SO (< 1.25 Hz) spectral power. When appropriate, sleep spindles were reported as fast (~14–16 Hz) or slow spindles (~11–13 Hz) according to papers methods. Less common terms were also extracted such as *spindle distribution* (Huupponen et al. 2002; Santamaria et al. 2000) (i.e., the spatial pattern of sleep spindles across the scalp, categorized by regions [frontal, central, parietal], providing a detailed mapping of spindle activity), *spindle laterality* (Santamaria et al. 2000) (i.e., using a measure of spindle amplitude differences between hemispheres) and *spindle diffuseness* (Huupponen et al. 2002) (i.e., the number of channels out of 6 where spindles are detected simultaneously and the spatial distribution of spindles across frontal, central, and parietal regions, providing a detailed scalp‐wide mapping of activity).

We extracted sex effect estimates for spindle and SWA outcomes (i.e., spindle density, sigma power and delta power) as mean differences with variance measures. Outcomes of the studies were categorized as normal sleeper, insomnia, or both, for analysis and reporting. Where key data were missing or insufficient to derive effect sizes, authors were contacted when possible; otherwise, studies were excluded. Key data were defined as group‐level summary statistics (e.g., means) with an accompanying measure of variance (e.g., SD, SE, or CI) and sample size. All relevant results for each outcome were sought.

### 2.7 Risk of Bias Assessment

Data extraction and risk of bias assessment were independently conducted by 2 reviewers (N.A.W. and A.P.) using the Newcastle‐Ottawa Scale (NOS) (Wells et al. 2000) for cross‐sectional studies, case–control studies, cohort studies, and the PEDro scale for randomized controlled trials (de Morton 2009). Any discrepancies in risk of bias assessments were resolved through consensus between the authors. Studies deemed to be at high risk of bias were excluded from meta‐analysis. The quality of included studies informed the synthesis and interpretation of our results.

### 2.8 Sleep Spindle and SWA Measures

We extracted means, standard deviations, and sample sizes from studies reporting EEG/PSG data on sleep spindle and SWA measures.

### 2.9 Data Analysis

Descriptive statistics were used to quantify and describe the results of our systematic review literature searches, screening, and study characteristics (e.g., outcomes). We expected clinical diversity and heterogeneity between studies resulting from variable samples (e.g., mean ages, proportion of men and women), study locations and settings. Studies with small sample sizes and large standard errors may also bias the results of pooled effect estimates in meta‐analysis (Deeks et al. 2024). Therefore, following the guidance of the Cochrane Handbook for Systematic Reviews of Interventions, we conducted inverse‐variance weighted random‐effects (DerSimonian and Laird 2015) meta‐analyses with Review Manager 5.41 software.

Individual Hedges *g* effect sizes/standardized mean differences between males and females in spindle and SWA were calculated in RevMan 5.41 with continuous outcome data from each paper eligible for meta‐analysis to account for differences in scales or processes between papers (Deeks et al. 2024).

Pooled data from meta‐analyses on sleep spindle density and SWA were displayed graphically using Forest plots.

Heterogeneity was assessed using *τ* ^2^, *χ* ^2^ (*α* = 0.10), and *I* ^2^ (low: 0%–30%, moderate: 30%–50%, substantial: 50%–75%, considerable: > 75%) (Higgins et al. 2021). Forest plots supported visual inspection (Higgins et al. 2021; Ryan 2016). When *I* ^2^ > 50%, sensitivity analyses were performed by sequentially excluding individual studies (Higgins et al. 2021; Anzures‐Cabrera and Higgins 2010).

### 2.10 Publication Bias

Publication bias was explored visually using effect size (Hedges g) by standard error funnel plots (Higgins et al. 2021). We did not conduct additional statistical tests (e.g., Egger's test, trim‐and‐fill) or assess other types of reporting bias (e.g., selective outcome reporting). In meta‐analyses with a small sample of studies (< 10 papers) per outcome, the power of these tests is too low to distinguish real asymmetry from chance (Higgins et al. 2021). As a result, Egger's tests for publication bias were not undertaken.

## 3 Results

### 3.1 Study Selection

Our systematic searches yielded 13,290 papers from PubMed, Embase, Scopus, and PsycInfo (Figure 1). After removing 3782 duplicates, 9506 papers were reviewed by title and abstract. From these, 9064 papers were excluded. Four hundred eighty‐six papers were deemed eligible or likely eligible and screened by full‐text, of which 224 were excluded after full screening. Citation searching among relevant but ineligible papers (Carrier et al. 2017; Baglioni et al. 2014), added 4 eligible papers, resulting in 257 papers meeting our inclusion criteria. Of these, 17% (43 papers) analysed sex‐based differences in spindle or SWA: 33 on normal sleepers (NS) only, 2 on insomnia (IN) only, and 8 on both populations (Tables 1, 2, 3). Attrition and acknowledgement of sex‐based difference per population study (NS, IN, mixed) can be found in the Supporting Information.

**FIGURE 1:**
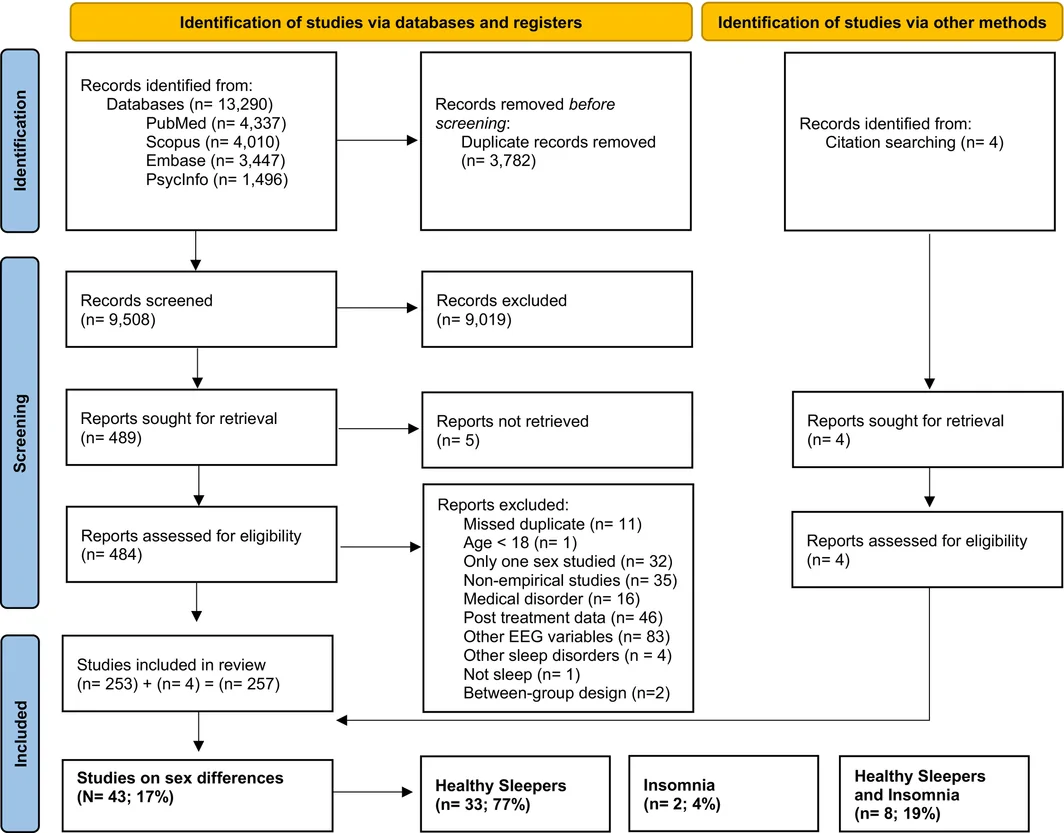
Flow diagram for systematic review and Meta‐analysis.

**TABLE 1:** Risk of bias summary plot according to the Newcastle‐Ottawa scale for cross sectional, cohort, case–control studies, and randomized controlled trial studies.

Type of study	Group	Study	Representative	Sample size	Non‐respondents	Ascertainment	Comparability	Outcome	Statistical test	Total risk of bias
Cross sectional	Insomnia	Bader et al. (2013)	3	2	3	1^**^	0	2^**^	1	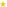
		Hall et al. (2000)	2	2	3	2	1^**^	3	0	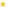
		Armitage (1995)	4	3	3	N/A	1	3^*^	1	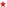
	Normal Sleepers	Armitage, Hoffmann, Fitch, et al. (2000)	2	2	3	2	2	2^**^	1	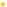
		Armitage, Hoffmann, Trivedi, and Rush (2000)	2	2	3	2	1^**^	2^**^	0	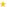
		Bové et al. (1994)	4	2	3	3	2	2^**^	0	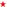
		Carrier et al. (2001)	1	3	3	1^**^	1^**^	2^**^	1	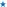
		Carrier et al. (2011)	2^*^	2	3	3	1^**^	2^**^	0	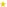
		Colrain et al. (2009)	2	2	3	2	1^**^	1^**^	1	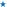
		De Gennaro et al. (2017)^a^	2	2	3	1^**^	1^**^	2^**^	0	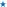
		Dijk et al. (1989)	2	2	3	3	1^**^	2^**^	0	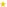
		Ehlers and Kupfer (1997)	2	2	3	3	1^**^	2^**^	0	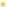
		Fukuda et al. (1999)	4	3	3	3	0	4	1	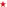
		Gaillard and Blois (1981)	2	2	1	3	0	2^**^	1	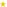
		Goldschmied et al. (2014)	2	3	3	1^**^	1^**^	2^**^	1	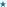
		Gorgoni et al. (2016)	1	3	3	1^**^	1^**^	2^**^	1	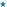
		Hertenstein et al. (2018)	1	2	3	1^**^	1^**^	2^**^	0	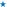
		Huupponen et al. (2002)	2	2	3	2	1^**^	4	1	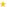
		Landolt and Gillin (2005)	2	3	3	1^**^	1^**^	2^**^	0	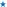
		Latreille et al. (2011)	2	3	3	1^**^	0	2^**^	0	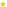
		Latta et al. (2005)	2	2	3	1^**^	0	2^**^	0	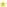
		Luca et al. (2015)	1	3	3	1^**^	1^**^	2^**^	1	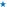
	
		Massicotte‐Marquez et al. (2005)	1	3	3	2	0	2^**^	0	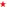
		Munch et al. (2007)	2	2	3	2	0	3	0	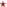
		Ondze et al. (2003)	2	2	3	1^**^	0	2^**^	0	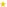
		Plante et al. (2013)	2	2	3	1^**^	1^**^	2^**^	0	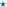
		Plante et al. (2012)	2	2	3	1^**^	1^**^	2^**^	1	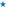
		Richards et al. (2013)	2	3	3	2	0	2^**^	1	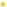
		Ritter et al. (2018)	2	1	1	1^**^	1^**^	1^**^	1	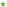
		Santamaria et al. (2000)^b^	2	3	3	1^**^	0	2^**^	0	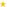
	Ujma et al. (2014)	3	2	3	3	1^**^	2^**^	1	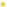
	Insomnia and normal sleepers	Benbir Şenel et al. (2021)	2	3	3	1^**^	1^**^	1^**^	0	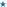
		Buysse et al. (2008)	2	3	3	1^**^	1^**^	2^**^	0	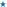
		Lunsford‐Avery et al. (2021)	2	2	3	1^**^	1^**^	1^**^	0	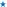
		Shao et al. (2022)	2	3	3	1^**^	1^**^	2^**^	0	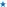
		Spiegelhalder et al. (2012)	3	3	3	1^**^	0	2^**^	0	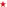
		Svetnik et al. (2017)	3	3	3	1^**^	0	1^**^	1	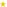
Wu et al. (2013)		3	3	3	1^**^	1^**^	2^**^	0	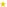

Type of study	Group	Study	Representative	Selection	Ascertainment	Demonstration	Comparability	Outcome	Follow‐up	Adequacy	Total risk of bias
Cohort	Normal sleepers	Coelho et al. (2025)	1, 2	1	1, 2	1	1, 2	1, 2	1	1, 2	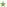
		Djonlagic et al. (2021)	1	1	1, 2	1	1	1	1	4	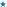
		Rosinvil et al. (2021)	2	1	1	2	1	4	1	4	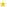
	Insomnia and normal sleepers	Krystal et al. (2002)	1, 2	1	1, 2	1	1, 2	1, 2	1	1, 2	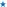

Type of study	Group	Study	Eligibility	Randomized	Allocation	Baseline	Blinding subjects	Blinding therapists	Blinding assessors	Respondents	Treatment	Statistics	Measures	Total risk of bias
RCT	Insomnia and normal sleepers	Jones et al. (2024)	1	1	2	1	2	2	2	1	1	1	1	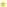

*Note:* 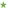 Very Good Studies: 9–10 points. 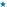 Good Studies: 7–8 points. 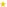 Satisfactory Studies: 5–6 points. 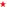 Unsatisfactory Studies: 0–4 points.

* One point from the Newcastle‐Ottawa scale.

** Two points from the Newcastle‐Ottawa scale.

^a^ Cross‐sectional analysis of case–control study.

^b^ Cross‐sectional analysis of cohort study.

### 3.2 Risk of Bias Assessment

Among the 43 studies reviewed, most exhibited low to moderate risk of bias with satisfactory to good quality. Fourteen percent showing high‐risk concerns according to the Newcastle‐Ottawa Scale (See Table 1). The primary risk of bias resulted from inadequate sample size justification, missing data on non‐respondents, and the absence of effect size measures (e.g., confidence intervals, *p*‐values). These issues suggest that many studies were potentially underpowered, affecting the reliability of their findings. One study assessed with the PEDro Scale had fair‐to‐good study quality, with risk of bias arising from lack of reporting about participant or examiner blinding or allocation concealment (Cashin and Mcauley 2019).

### 3.3 Findings From the Systematic Review

#### 3.3.1 Normal Sleepers

Thirty‐three of the included papers directly examined sex‐based differences in brain oscillations during sleep (see Table 2). Out of the 33 studies evaluating sex‐based differences, 17 reported on spindle activity, 23 reported on SWA characteristics, and 3 reported specifically on SO characteristics. It is important to note that while all the papers included in our review examined sex‐based differences for at least one type of brain oscillation, they did not do so systematically across all oscillatory measures. For example, some studies assessed sex‐based differences in sigma power but not in delta power. Nevertheless, these studies were included in both sections of our review to evaluate the effects of insomnia on each brain oscillation, regardless of whether sex‐based differences were specifically analysed for every measure.

##### 3.3.1.1 Spindle Activity

Most studies (55%) investigating brain oscillations in NS did not examine sex‐based differences. Seventeen research papers explored sex‐based differences in spindle characteristics in NS, representing a total of 3587 participants (mean age = 41.4 ± 18.6, median group size = 35, range = [10, 1595]; see Table 2) and 9 spindle characteristic outcomes (sigma power, spindle density, spindle frequency, spindle peak frequency, spindle amplitude, spindle duration, spindle laterality, spindle distribution, and spindle diffuseness). There is some evidence that females exhibited higher spindle density (25% of papers), spindle amplitude (67% of papers), and sigma power (67% of papers) compared to male NS. Measures of relative sigma power showed fewer differences.

###### 3.3.1.1.1 Spindle (Event‐Based Detection)

The most commonly reported domains were spindle density (*k* = 8). Spindle detection methods varied between papers (Table 2). Out of 8 papers specifically reporting on spindle density, 38% (*k* = 3) reported significant sex‐based differences, while 62% (*k* = 5) did not (Huupponen et al. 2002; Santamaria et al. 2000; Gorgoni et al. 2016; Bové et al. 1994; Ritter et al. 2018). Of the 3 papers finding differences between sexes in spindle density, 67% (*k* = 2) found higher density in females (Djonlagic et al. 2021; Gaillard and Blois 1981) (1 (Djonlagic et al. 2021) specific to fast spindles, and 1 specifically only during NREM2 (Gaillard and Blois 1981)), and 33% (*k* = 1) found higher density in males (Ujma et al. 2014). The majority of papers not finding sex‐based differences in spindle density (60%; *k* = 3) only analysed spindles during NREM2 (Huupponen et al. 2002; Santamaria et al. 2000; Bové et al. 1994).

Other spindle characteristic outcomes were less commonly reported across studies. Two articles (Ritter et al. 2018; Coelho et al. 2025) reported on spindle frequency (Hz), 1 found no sex‐based differences (Ritter et al. 2018), while the other indicated males had a higher frequency than females (Coelho et al. 2025). Three studies reported on spindle amplitude; 1 (50%) (Santamaria et al. 2000) reported no sex‐based differences, while the others found females had higher spindle amplitudes, specifically in the fast spindle range (F3) (Ujma et al. 2014; Coelho et al. 2025). One of the 2 found the same for the slow spindle range (C3) (Coelho et al. 2025). The same study was the sole paper that looked at spindle duration (Ujma et al. 2014), finding that males had greater fast spindle duration (13–15 [12.5–15.38] Hz) compared to females in Oz. Two studies on spindle distribution (100%) reported higher distribution in females (Huupponen et al. 2002; Santamaria et al. 2000). One specifically found this relationship to be true in the left frontal area but not central or occipital (Huupponen et al. 2002). There were no sex‐based differences in spindle laterality (Santamaria et al. 2000) and spindle diffuseness (Huupponen et al. 2002) (*k* = 1).

###### 3.3.1.1.2 Sigma Spectral Power

Beyond spindle activity based on event detection, some studies studied spindle activity using power spectral analysis referred to as sigma power (*k* = 9). Five (56%) (Hertenstein et al. 2018; Djonlagic et al. 2021; Carrier et al. 2001; Dijk et al. 1989; Luca et al. 2015) of 9 (Ondze et al. 2003; Richards et al. 2013; Plante et al. 2013; Ehlers and Kupfer 1997) studies reporting on sex‐based differences in sigma power observed higher absolute or relative sigma power in females on central channels, including 1 reporting this true for only fast sigma on central electrodes (14–16 Hz (Hertenstein et al. 2018)), and 1 for only slow sigma (11.25–13 Hz (Luca et al. 2015)) on central electrodes, and 1 finding this only when considering relative slow sigma power (11.25–13 Hz on C4 (Luca et al. 2015)). One of the studies that did not find sex‐based differences indicated that both males and females showed similar age‐related reductions in sigma power (Ehlers and Kupfer 1997). Concerning spindle morphology, 1 article on spindle peak frequency (Hz) using power spectral analysis found females had higher peak frequencies, 1 confirming this for both fast and slow spindles (Ujma et al. 2014).

##### 3.3.1.2 Slow Wave Activity

Twenty‐four research papers explored sex‐based differences in SOs (< 1.25 Hz) and SWA (0.25–4.5 Hz) characteristics in NS. The 24 studies reported a total of 3996 participants (mean age = 42.30 ± 18.30, median group size = 31, range 16 to 1595; see Table 2) and 8 differing SWA characteristic outcomes either based on power spectral analyses in the delta (~0.25–4 Hz) or SO (< 1.25 Hz) frequency range or based on event detection (SW density, SW amplitude, SW frequency, SW slope, SW incidence, SW positive phase duration and SO event density). There were inconsistent findings on the presence of sex‐based differences in SWA. SW amplitude appeared to be higher in females (75% of papers). Females also tended to exhibit higher absolute delta power than males (48% of papers), although half of the studies showed no difference in absolute delta power. Measures of relative delta power showed fewer differences.

###### 3.3.1.2.1 Slow Wave and Slow Oscillation (Event‐Based Detection)

The most commonly reported domain was SW amplitude (*k* = 4). SW detection methods varied between papers (see Table 2). For SW amplitude, 75% (3 of 4 studies (Armitage, Hoffmann, Trivedi, and Rush 2000; Carrier et al. 2011; Rosinvil et al. 2021)) found higher SW amplitude in females, while 1 did not (Armitage, Hoffmann, Fitch, et al. 2000). Furthermore, 1 study found an age‐by‐sex interaction, showing that SW amplitude was significantly higher in older adults than in younger ones, with a steeper decline observed in older men (Rosinvil et al. 2021). Two studies reported a steeper SW slope in females, with 1 finding this relationship regardless of age (Rosinvil et al. 2021), and 1 finding that females had a steeper SW slope across the lifespan (Armitage, Hoffmann, Trivedi, and Rush 2000). SW frequency and SW positive and negative phase duration appear higher in males (Carrier et al. 2011) (*k* = 1). Of the 3 studies on SW density, 1 paper did not find sex‐based differences (Armitage, Hoffmann, Fitch, et al. 2000), 1 found greater SW density in females (Carrier et al. 2011), while the other found that with adapted criteria, older adults showed lower SW density than younger participants, regardless of sex (Rosinvil et al. 2021). There was no standalone sex effect, but a sex‐by‐age interaction emerged—older females retained stronger SW density, compared to older men. This suggests that while aging reduces SW features overall, females tend to maintain greater SW density later in life. The sole study on SO density (< 1.25 Hz) found that females exhibited higher SO density than males (Djonlagic et al. 2021).

###### 3.3.1.2.2 Delta and Slow Oscillation Spectral Power

The most frequently examined domain was delta spectral power (*k* = 21), followed by SO power (*k* = 3). Among the 21 studies on delta power, 9% (*k* = 2) reported no sex‐based differences in relative delta power (Luca et al. 2015; Latta et al. 2005), and 48% (*k* = 10) reported no differences in absolute delta power (Ondze et al. 2003; Richards et al. 2013; Armitage, Hoffmann, Trivedi, and Rush 2000; Armitage, Hoffmann, Fitch, et al. 2000; De Gennaro et al. 2017; Goldschmied et al. 2014; Landolt and Gillin 2005; Latreille et al. 2011; Munch et al. 2007; Plante et al. 2012; Armitage 1995). Conversely, 9 (43%) studies found higher absolute delta power in females (Hertenstein et al. 2018; Carrier et al. 2001; Dijk et al. 1989; Latta et al. 2005; Armitage 1995; Colrain et al. 2009; Fukuda et al. 1999; Massicotte‐Marquez et al. 2005; Jones et al. 2024), 1 (5%) in relative delta power (Djonlagic et al. 2021), and 1 (5%) study (Ehlers and Kupfer 1997) reported greater age‐related reductions in absolute delta power in males within the 0.75–4.5 Hz range, but not when looking at mean delta power density in the broader 0.5–4.5 Hz range. Notably, 1 study (Luca et al. 2015) reported that this sex‐based difference was limited to the SO range (0.75–1.25 Hz) and not observed in the 1.25–4.5 Hz range, while another (Massicotte‐Marquez et al. 2005) found it only during the first 3 NREM cycles. In contrast, 1 paper (Latta et al. 2005) found that when delta power was normalized to the percentage of delta activity in REM, females exhibited lower delta activity than males. Of the 10 studies (40%) that did observe sex‐based differences in SWA, most focused on absolute delta power, rather than relative or normalized measures. One study (Colrain et al. 2009) noted that elevated absolute delta power in females was most pronounced in the Pz and O_2_ channels. One study on SW incidence reported no differences between sex (Armitage, Hoffmann, Fitch, et al. 2000). Finally, all 3 studies examining absolute and relative SO power (< 1.25 Hz) (Hertenstein et al. 2018; Djonlagic et al. 2021; Luca et al. 2015) reported higher SO power in females compared to males (100%).

**TABLE 2:** Study characteristics of normal sleepers.

Study	*N*	Age	*F* (%)	Country	Design	Adaptation night?	Stage	Channel	Brain oscillation	Method used	Sex‐based difference?	Difference	Hormone
(Armitage 1995)	22	25.2 ± 4.0	50	USA	Cross‐Sectional	Yes	NREM	C3, C4 (referenced to linked earlobes)	Absolute Delta power	Power Spectral Analysis [0.5–4 Hz] (Massicotte‐Marquez et al. 2005) 250 Hz	Yes (*F*_1,20_ = 6.24, *p* < 0.02)	*F* > *M*	No
(Armitage, Hoffmann, Fitch, et al. 2000)	23	28.4 ± 5.9	35	USA	Cross‐Sectional	Yes	NREM	C3, C4 (referenced to linked earlobes)	SW Amplitude	Period‐Analysis Algorithm [0.5–4 Hz] (Jones et al. 2024)	No (*p* = 0.65)		All females on birth control (oral)
									SW Incidence		No (*F* < 1)		
									Absolute Delta Power	Power Spectral Analyses (Pigeau et al. 1981) 250 Hz	No (*p* > 0.05)		
(Armitage, Hoffmann, Trivedi, and Rush 2000)	55	29.5 ± 5.6	42	USA	Cross‐Sectional	Yes	NREM	C3, C4 (referenced to linked earlobes)	SW Amplitude	Period‐Analysis Algorithm [0.5–4 Hz] (Jones et al. 2024) 250 Hz	Yes	*F* > *M*	No
									SW Slope		Yes (*p* = 0.0003)	*F* > *M* steeper	
(Bové et al. 1994)	11	40.1 ± 16.0		Spain	Cross‐Sectional	No	NREM2	C3‐A1, C4‐A2	Spindle Density	Manual Detection Sampling Rate and Frequency Range^d^	No (*t* = 0.45, no *p*)		Menstrual cycle phase
(Carrier et al. 2001)	100	20–60^a^	47	USA	Cross‐Sectional	Yes	NREM	C4‐A1/A2	Absolute Delta Power^i^	Power Spectral Analysis^d^ (FFT) 128 Hz	Yes^f^	*F* > *M*	No
									Absolute Sigma Power^i^		Yes^f^	*F* > *M*	
(Carrier et al. 2011)	87	36.1 ± 14.8		Canada	Cross‐Sectional	Yes	NREM	Fp1, F3, C3, P3, O1 (referenced to linked earlobes)^b^	SW Density	Fixed Threshold Automatic Detection [0.3–4 Hz] (Dang‐Vu et al. 2008) 256 Hz	Yes (*F*_1,83_ = 4.1; *p* < 0.05)	*F* > *M* steeper	Hormone regulated females (e.g., no birth control or menopause)
									SW Positive and Negative Phase Duration		Yes (*F*_1,83_ = 4.6; *p* = 0.03)	*M* > *F*	
									SW Frequency		Yes (*F*_1,83_ = 4.3; *p* = 0.04)	*M* > *F*	
									SW Amplitude		Yes (*F*_1,83_ = 5.5; *p* = 0.02)	*F* > *M*	
(Coelho et al. 2025)	80	40.9 [23.1–53.0]^h^	50	France	Cohort	No	NREM2	C3‐A2, F3‐A2	Spindle Amplitude [Slow: 10.5–13 Hz]	Automatic Detection With Adaptative Amplitude Threshold (Armitage and Roffwarg 1992) 100 Hz	Yes (*p* = 0.025)	*F* > *M*	No
											Yes (*p* = 0.013)	*F* > *M*	
									Spindle Amplitude [Fast: 13.1–17.5 Hz]				
									Spindle Frequency		Yes (*p* = 0.020)	*M* > *F*	
(Colrain et al. 2009)	42	51.1 ± 9.7	55	USA	Cross‐Sectional	Yes	NREM	Fz, FCz, Cz, CPz, Pz^g^, O2^g^‐A1 or A2^b^	Absolute Delta Power	Power Spectral Analysis (FFT) [0.3–4 Hz] (Press and Rybicki 1989) 1000 Hz	Yes (*F*_1,70_ = 6.7, *p* = 0.012)	*F* > *M*	No
(De Gennaro et al. 2017)	20	70.4 ± 6.3	40	Italy	Cross‐Sectional	No	NREM	19 derivations‐A1/A2^b^	Absolute Delta Power Ratio SO (< 1 Hz)/Delta power	Power Spectral Analysis (FFT) [0.6–4 Hz] (Berthomier et al. 2007) 256 Hz	No^f^	.	No
(Dijk et al. 1989)	28	22.6 ± 2.0	54	Netherlands	Cross‐Sectional	Yes	NREM	C3‐A2, C4‐A1 (best of the 2 EEG lead)	Absolute Delta Power (Log‐transformed)	Power Spectral Densities (0.25 Hz bins) Frequency Range^d^ 64 Hz	Yes (*p* < 0.05)	*F* > *M*	Females not during menses and most on birth control (oral)
									Absolute Sigma Power (Log‐transformed)		Yes (*p* < 0.05)	*F* > *M*	
(Djonlagic et al. 2021)	1595	54–93^a^	52	USA	Cohort	No	NREM	C4	Spindle Density [Slow: 9–11 Hz]	Wavelet‐based Automatic Detection (Welch 1967) 100 Hz	No (*b* = 0.07; 95% CI = −0.02 to 0.17; *p* = 0.12).	.	No
									Spindle Density [Fast: 13–17 Hz]		Yes (*b* = −0.18; 95% CI = −0.24 to −0.08; *p* = 2 × 10^−4^).	*F* > *M*	
									Relative Sigma Power [12–15 Hz]	Power Spectral Analysis (FFT) (Press and Rybicki 1989)	Yes (*b* = −0.78; 95% CI = −0.87 to −0.70; *p* < 10^−15^)	*F* > *M*	
									Relative SO Power [0.5–1 Hz]		Yes (*b* = −0.78; 95% CI = −0.87 to −0.70; *p* < 10^−15^)	*F* > *M*	
									Relative Delta Power [1–4 Hz]		Yes (*b* = −0.78; 95% CI = −0.87 to −0.70; *p* < 10^−15^)	*F* > *M*	
							NREM3		SO Density	Automatic Detection With Adaptative Amplitude Threshold [0.5–1 Hz]	Yes (*b* = −0.71; 95% CI = −0.60 to −0.81; *p* < 10^−15^)	*F* > *M*	
(Ehlers and Kupfer 1997)	61	20–40^a^	46	USA	Cross‐Sectional	No	Whole‐night	C4‐A1/A2	Absolute Delta Power	Power Spectral Analysis [0.75–4.5 Hz] 128 Hz	Yes (*F*_1.31_ = 33.9, *p* < 0.0001).	Age reductions for *M* but not *F*	All females follicular phase
									Delta Power Density	Mean Power Density [0.5–4.5 Hz] (Mander et al. 2015)	No (*F*_1,57_ = 3.27, *p* < 0.07)		
									Absolute Sigma Power	Power Spectral Analysis [11–16 Hz]	No^f^	*M* = *F* age reductions	
(Fukuda et al. 1999)	16	61.9 ± 5.6	50	Japan	Cross‐Sectional	Yes	Whole‐night	C3‐A2	Absolute Delta Power 1 [0.5–2 Hz]	Power Spectral Analysis (FFT; cosine window tapering) 125 Hz	Yes (*p* < 0.01)	*F* > *M*	No
									Absolute Delta Power 2 [2–4 Hz]		Yes (*p* < 0.01)	*F* > *M*	
(Gaillard and Blois 1981)	10	33.0 ± 11.1	50	Switzerland	Cross‐Sectional	Yes	NREM2	C4‐T4	Spindle Density	Automatic Detection With Fixed Thresholds [11.6–17.2 Hz]. Sampling Rate^d^	Yes (*t* = 2.45, *p* < 0.05)	*F* > *M*.	No
							NREM3 & NREM4				No (both *p* < 0.1)		
(Goldschmied et al. 2014)	80	29.3 ± 6.0	50	USA	Cross‐ Sectional	Yes	NREM (1st cycle)	C3, C4, F3, F4, P3, P4, O1, O2 (referenced to linked earlobes)^b^	Absolute Delta Power	Power Spectral Analysis (FFT; Hanning window tapering)^d^ 256 Hz	No (*t* = −1.05, Cohen's *d* = 0.16 *p* = 0.47)		No
							NREM (2nd cycle)				No (*t* = −0.72, Cohen's *d* = 0.24, *p* = 0.3)		
	
							NREM (3rd cycle)				No (*t* = −0.65, Cohen's *d* = 0.14, *p* = 0.52)		
Gorgoni et al. 2016	15	70.8 ± 1.6	33	Italy	Cross‐Sectional	No	NREM	Pz‐A1/A2 and F3, F4‐A1/A2 (used channel with maximal event detected)^b^	Slow Spindle Density [11–13 Hz] Fast Spindle Density [13–15 Hz]	Bandpass‐Filtering Automatic Detection (Wamsley et al. 2012) 256 Hz	No^f^		No
(Hertenstein et al. 2018)	206	39.8 ± 12.9	59	Germany	Cross‐Sectional	Yes	NREM2	C3‐A2, C4‐A1	Absolute SO Power [0.1–1 Hz]	Power Spectral Analysis (FFT) (Ehlers and Havstad 1982) 200 Hz	Yes (*p* < 0.001, variance 7.7%)	*F* > *M*	No
									Absolute Delta Power [1–3.5 Hz]		Yes (*p* < 0.001, variance 9.4%)	*F* > *M*	
									Absolute Sigma Power 1 [12–14 Hz]		Yes (*p* < 0.001, variance 16.6%)	*F* > *M*	
									Absolute Sigma Power 2 [14–16 Hz]		Yes (*p* < 0.001, variance 13.1%)	*F* > *M*	
(Huupponen et al. 2002a)	40	22–49^a^	50	Finland	Cross‐Sectional	Yes	NREM2	Fp1‐A2, Fp2‐A1, C3‐A2, C4‐A1, O1‐A2, O2‐A1	Spindle Density	Fuzzy Automatic Detection [11.1–16 Hz] (Ferrarelli et al. 2007) 200 Hz or 256 Hz	No (*p* = 0.10)	.	No
									Relative Spindle Distribution (topography)		Yes (*p* = 0.026)	*F* > *M* (for Fp1‐A2)	

									Relative Spindle Diffuseness		No (*p* = 0.008)	.	
(Jones et al. 2024)	221	29.9 ± 6.9	48	USA	RCT	Yes	NREM3	C3‐A2	Absolute Delta Power	Power Spectral Analysis (FFT) [0.5–4.5 Hz] 182 Hz	Yes (*β* = −1516.99; *p* < 0.001).	*F* > *M*	No
(Landolt and Gillin 2005)	16	41.1 ± 2.1	50	USA	Cross‐Sectional	Yes	NREM	F4‐C4, C4‐P4, P4‐O2, C3‐A2^b^	Absolute Delta Power	Power Spectral Analyses (FFT, cosine tapering window) [0.77–4.5 Hz] 512 Hz	No^f^	.	No
(Latreille et al. 2011)	30	59.6 ± 10.9	33	Canada	Cross‐Sectional	No	NREM	F3, C3, P3, O1 (referenced to linked earlobes)^b^	Absolute Delta Power	Power Spectral Analyses (FFT, cosine tapering window) [0.25–4 Hz] 256 Hz	No [effect size − *f* = 0.11]	.	No
(Latta et al. 2005)	20	61.0 ± 6.5	50	USA	Cross‐Sectional	Yes	NREM	Cz‐A1	Absolute Delta Power	Power Spectral Analyses (FFT, hamming window) [0.5–4 Hz] Sampling rate^d^	Yes (*t* = 0.035)	*F* > *M*	No hormonal therapies or menopause
									Relative Delta Power (% total power)		No (*t* = 0.2897)	.	
									Normalized Delta Power (% of delta activity in REM)		Yes (*t* = 0.0439)	*M* > *F*	
(Luca et al. 2015)	1147	56.8 ± 10.4	51	Switzerland	Cross‐Sectional	No	NREM	C3‐M2	Relative SO Power [0.75–1.25 Hz]	Power Spectral Analysis (FFT; Hamming window) 256 Hz	Yes (*p* < 0.001)	*F* > *M*	No
									Relative Delta Power [1.25–4.5 Hz]		No^f^		
									Relative Sigma Power [Slow:11.25–13 Hz]		Yes (*p* < 0.001)	*F* > *M*	
									Relative Sigma Power [Fast: 13.25–15 Hz]		No^f^		
(Massicotte‐Marquez et al. 2005)	28	66.3 ± 6.9	25	Canada	Cross‐Sectional	No	NREM	C3‐A2	Absolute Delta Power	Power Spectral Analysis (FFT; cosine window tapering) [0.75–4 Hz] 256 Hz	No^f^		No
							NREM (first 3 cycles)				Yes^f^	*F* > *M*	
(Munch et al. 2007)	31 (YA:16 OA:15)	YA: 25.3 ± 3.3 OA: 65.1 ± 5.6	YA: 50 OA: 47	Switzerland	Cross‐Sectional	Yes	NREM	Fz, Cz, Pz, Oz^b^	Absolute Delta Power	Power Spectral Analysis (FFT; cosine window tapering) [0.5–1.25 Hz] 128 Hz	No (*p* > 0.1)		No
(Ondze et al. 2003)	18	33.1 ± 11.7	61	France	Cross‐Sectional	Yes	NREM	C3‐A2, C4‐A1	Absolute Sigma Power [13–17 Hz]	Power Spectral Analysis (FFT; hamming window tapering) (Press and Rybicki 1989) 128 Hz	No^f^		No
									Absolute Delta Power [0.5–2 Hz]		No^f^		
(Plante et al. 2012)	30	25.4 ± 8.5	63	USA	Cross‐Sectional	No	NREM	185 channels (hd‐EEG)^b^	Absolute Delta Power	Power Spectral Analysis (FFT; hamming window tapering) (Press and Rybicki 1989) [1–4.5 Hz] 500 Hz	No (*p* = 0.28)		No
(Plante et al. 2013)	30	25.4 ± 8.5	63	USA	Cross‐Sectional	No	NREM	Average 173 channels (hd‐EEG)	Absolute Sigma Power (spindle range activity)	Power Spectral Analysis [11–15 Hz] 500 Hz	No (*p* = 0.44)		No
(Richards et al. 2013)	43	30.4 ± 8.2	49	USA	Cross‐Sectional	Yes	NREM	C3‐A1/A2	Absolute Delta Power [1–4 Hz]	Power Spectral Analysis (FFT) (Press and Rybicki 1989) 256 Hz	No (*F* = 0.15, *p* = 0.697)		All females studied during follicular phase
									Absolute Sigma Power [12–15 Hz]		No (*F* = 3.04, *p* = 0.085)		
(Ritter et al. 2018)	25	32.3 ± 7.0	68	Germany	Cross‐Sectional	No	NREM2	F3‐M2, F4‐M1, C3‐M2, C4‐M1^b^	Spindle Density [10–16 Hz]	Automatic Detection With Fixed Thresholds Sampling Rate^d^	No^f^		No
									Spindle Frequency		No^f^		
								C4	Spindle Density [Fast: 13–16 Hz]		No (*b* = 0.824, *t* = 1.682, *p* = 0.110)		
(Rosinvil et al. 2021)	77 (YA:38 OA:39)	YA: 22.7 ± 2.4 OA: 59.6 ± 5.4	YA: 50 OA: 51	Canada	Cohort	No		C3, C4 (referenced to linked earlobes)	SW Density	Fixed Threshold Automatic Detection (Dang‐Vu et al. 2008) [0.3–4 Hz] 256 Hz	Yes (*F*_1,73_ = 25.7, *p* < 0.0001, ES = 0.26)	OA < YA (stronger effect in *M* [*F* > *M*] with adapted criteria)	No
									SW Amplitude	Data‐Driven Automatic Detection (age and sex adapted amplitude criterion)	Yes (*F*_1,73_ = 160.8, *p* < 0.0001, ES = 0.69)	OA < YA (stronger effect in *M* [*F* > *M*])	
									SW Slope		Yes (*F*_1,73_ = 8.5, *p* = 0.005, ES = 0.10)	*F* > *M* (regardless of age)	
(Santamaria et al. 2000)	18	69 ± 5.7	61	Spain	Cross‐Sectional	Yes	NREM2 (2 10‐min segment)	Fp1, Fp2, C3, C4, P3, P4, O1, O2^b^	Spindle Density	Manual Detection Sampling Rate and Frequency Range^d^	No^f^		No
									Spindle Amplitude		No^f^		
		
									Spindle Laterality		No^f^		
		
									Spindle Distribution		Yes (*p* = 0.079)	*F* > *M*	
(Ujma et al. 2014)	160	29.7 ± 10.7	45	Multicentre	Cross‐Sectional	Yes	NREM	Fp1, Fp2, F3, F4, Fz, F7, F8, C3, C4, Cz, P3, P4, T3, T4, T5, T6, O1, O2	Fast Spindle Density	Adapted Automatic detection (Individual Adjustment Method) (Spiegelhalder et al. 2012) [9–16 Hz] 249,250, & 1024 Hz	Yes [*t* = P3 (2.00), P4 (2.00), O1 (2.35), O2 (3.08)] [*p* = P3 (0.04), P4 (0.04), O1 (0.02), O2 (0.002)]	*M* > *F*	No
									Fast Spindle Amplitude		Yes [*t* = F3 (2.18), F4 (2.66), Fz (2.39), C3 (2.55), C4 (2.83), Cz (3.55), P3 (2.99), P4 (2.66), T6 (2.07), O1 (2.51), O2 (2.14)] [*p* = F3 (0.03), F4 (0.008), Fz (0.02), C3 (0.01), C4 (0.005), Cz (0.0005), P3 (0.003), P4 (0.009), T6 (0.04), O1 (0.01), O2 (0.03)]	*F* > *M*	
									Slow Spindle Peak Frequency		Yes (*t* = 2.82, *p* = 0.005)	*F* > *M*	
									Fast Spindle Peak Frequency		Yes (*t* = 4.13, *p* = 0.00006)	*F* > *M*	
								O2	Fast Spindle Duration		Yes (*t* = 2.57, *p* = 0.01)	*M* > *F*	

*Note:* NREM‐N2 + N3 + N4 (in papers older than 2007).

Abbreviations: *F*, female; *M*, male; OA, older adult; YA, young‐adult.

^a^ No mean or SD provided.

^b^ Unspecified channel reported for statistical analyses.

^c^ No SD provided.

^d^ Unspecified.

^e^ Calculated from data provided.

^f^ No statistics provided.

^g^ Especially prominent.

^h^ Quartiles.

^i^ Bins self‐defined rather than spectral bands.

#### 3.3.2 Individuals With Insomnia

There were very few studies on brain oscillations in insomnia disorder. Seventeen studies on persons with insomnia only were eligible for the systematic review (See Table 3). Most studies (88%) investigating brain oscillations in IN did not examine sex‐based differences. Of those 17 papers, 2 directly compared sex‐based differences in brain oscillations during sleep. All studies reported on primary insomnia using DSM‐IV criteria (i.e., difficulties falling and/or staying asleep for at least 1 month).

##### 3.3.2.1 Spindle Activity

One paper explored sex‐based differences in spindle characteristics in those with insomnia. They reported a total of 45 participants (mean age = 45.1 ± 7.5; Table 3) and 1 spindle characteristic outcome (i.e., sigma power). There is some evidence that sigma power in insomnia is not significantly affected by sex. No studies examined sex‐based differences in spindle characteristics in insomnia.

###### 3.3.2.1.1 Spindle (Event‐Based Detection)

No studies to date have examined sex‐based differences in spindle characteristics among persons with insomnia using event detection.

###### 3.3.2.1.2 Sigma Spectral Power

A single study was conducted on sex‐based difference in sigma power in IN (Bader et al. 2013). Bader et al. (2013) delved into the investigation of potential sex‐based differences in sigma power (12–16 Hz) during NREM 2 and 3 phases among individuals with insomnia. Following an initial adaptation night, the researchers reported no significant disparities between sexes, though specific statistical data were not provided within a participant pool comprising mostly females (76%).

##### 3.3.2.2 Slow Wave Activity

Two research papers explored potential sex disparities in SWA characteristics in individuals experiencing insomnia, representing a total of 59 participants (mean age = 47.0 ± 8.7, median group size = 29.5, range 14 to 45; Table 3) and 1 SWA characteristic outcome (delta power). There is some evidence that delta power in insomnia is not significantly affected by sex. No studies examined sex‐based differences in SW and SO characteristics or SO power in insomnia.

###### 3.3.2.2.1 Slow Wave and Slow Oscillation (Event‐Based Detection)

No studies to date have examined sex‐based differences in SW or SO characteristics among individuals with insomnia using event detection.

###### 3.3.2.2.2 Delta and Slow Oscillation Spectral Power

The sole reported domain was delta power (*k* = 2). Two papers investigated sex‐based differences in delta spectral power but found no significant results regarding absolute (Bader et al. 2013; Hall et al. 2000), nor relative delta power (Bader et al. 2013). However, neither study reported statistical data. Both papers adhered to the DSM‐IV criteria for insomnia disorder, conducted the research following an adaptation night, and tested absolute delta power. However, Bader et al. and Hall et al. employed distinct definitions for delta power frequency (0.1–3.5 Hz and 0.5–4 Hz, respectively). No studies to date have examined sex‐based differences in SO power among persons with insomnia using power spectral analysis.

**TABLE 3:** Study characteristics of insomnia.

Study	*N*	Age	*F* (%)	Country	Design	Insomnia criteria	Adaptation night?	Stage	Channel	Brain oscillation	Method used	Sex‐based difference?	Difference	Hormone
(Bader et al. 2013)	45	45.1 ± 7.5	76	Switzerland	Cross‐Sectional	DSM‐IV Primary Insomnia	Yes	NREM	C3‐A2	Relative and Absolute Sigma Power	Power Spectral Analysis (FFT) [12–16 Hz] (Press and Rybicki 1989) 512 Hz	No^f^	.	No
										Relative and Absolute Delta power	[0.1–3.5 Hz]	No^f^	.	
(Hall et al. 2000)	14	53 ± 12	64	USA	Cross‐Sectional	DSM‐IV Primary Insomnia	Yes	NREM	C3‐A2 or C4‐A1	Absolute Delta Power	Power Spectral Analysis (FFT) [0.5–4 Hz] 128 Hz	No^f^	.	No

*Note:* NREM‐N2 + N3 + N4 (in papers older than 2007).

Abbreviations: *F*, female; *M*, male; OA, older adult; YA, young‐adult.

^a^ No mean or SD provided.

^b^ Unspecified channel reported for statistical analyses.

^c^ No SD provided.

^d^ Unspecified.

^e^ Calculated from data provided.

^f^ No statistics provided.

^g^ Especially prominent.

^h^ Quartiles.

^i^ Bins self‐defined rather than spectral bands.

**TABLE 4:** Study characteristics of insomnia and normal sleepers.

Study	*N*	Age	*F* (%)	Country	Design	Insomnia criteria	Adaptation night?	Stage	Channel	Brain oscillation	Method used	Group difference	Sex‐based difference	GxS difference?	Hormones
(Benbir Şenel et al. 2021)	40 [IN = 20, NS = 20]	IN:52.2 ± 8.6 NS:50.4 ± 7.8	IN: 70 NS: 65	Turkey	Cross‐Sectional	ICSD‐3 Paradoxical Insomnia	No	NREM2	C3, F4	Spindle Density	Semi‐Automatic Program Sampling Rate and Frequency Range^d^	Yes (*p* = 0.003) NS > IN	No^f^ *F* = *M*	.	No
										Spindle Density [Slow]		Yes (*p* = 0.005) NS > IN	.	.	
										Spindle Density [Fast]		Yes (*p* = 0.002) NS > IN	.	.	
										Spindle Duration		No (*p* = 0.289) NS = IN	No^f^ *F* = *M*	.	
										Spindle Duration [Slow]		No (*p* = 0.359) NS = IN	.	.	
										Spindle Duration [Fast]		No (*p* = 0.239) NS = IN	.	.	
										Spindle Frequency		No (*p* = 0.387) NS = IN	No^f^ *F* = *M*	.	
										Spindle Frequency [Slow]		No (*p* = 0.303) NS = IN	.	.	
										Spindle Frequency [Fast]		No (*p* = 0.573) NS = IN	.	.	
(Buysse et al. 2008)	73 [IN = 48, NS = 25]	IN:30.8 ± 7.2 NS:30.6 ± 7.4	IN: 60 NS: 60	USA	Cross‐Sectional	DSM‐IV Primary Insomnia + PSQI score > 5	Yes	NREM (first 4 cycles)	C3, C4‐A1/A2	Absolute Delta Power	Power Spectral Analysis (FFT; hamming window tapering) [0.5–4 Hz] 256 Hz	No (*p* = 0.92) NS = IN	No (*p* = 0.08) *F* = *M*	Yes (*p* = 0.01) FIN > FNS	No
										Relative Delta Power		No^f^ NS = IN	No^f^ *F* = *M*	Yes (*F* = 4.04, *p* = 0.05) MNS > FNS	
										Absolute Sigma Power	[12–16 Hz]	No (*p* = 0.50) NS = IN	No (*p* = 0.11) *F* = *M*	Yes (*p* = 0.04) FIN > FNS	
(Krystal et al. 2002)	50 [IN_sub_ = 12IN_obj_ = 18NS = 20]	IN_sub_: 56.1 ± 11.7 IN_obj_: 54.3 ± 9.9 NS:53.5 ± 10.4	IN_Sub_: 67 IN_Obj_: 61 NS: 60	USA	Cohort	DSM‐III‐R Sleep‐maintenance Insomnia + mean WASO > 60 min for 1 week	No [at home PSG]	NREM	C3‐A2	Relative Delta Power	Power Spectral Analysis (FFT; Hanning window tapering) [0.5–3.5 Hz] 128 Hz	Yes (*F* = 8.20, *p* < 0.003) NS > IN_sub_	No^f^ *F* = *M*	.	No
												No^f^ NS = IN_obj_	No^f^ *F* = *M*		
										Absolute Delta Power		No^f^ NS = IN_sub_ & IN_obj_	No^f^ *F* = *M*		
										Relative and Absolute Sigma Power	[12.5–16 Hz]	Yes (*F* = 16.30, *p* < 0.0007) NS < INS_sub_	No^f^ *F* = *M*	*F* = *M* but older age, lower relative power (*F* = 4.1, *p* < 0.05)	
												Yes (*F* = 3.70, *p* < 0.08) IN_sub_ > IN_obj_	No^f^ *F* = *M*	.	
												Yes (*F* = 6.00, *p* < 0.03) NS < IN_obj_	No^f^ *F* = *M*	.	
(Lunsford‐Avery et al. 2021)	104 [IN = 51, NS = 53]	IN:42.7 ± 16.5 NS:40.7 ± 15.6	IN: 55 NS: 55	USA	Cross‐Sectional	DSM III/IV Chronic Primary Insomnia	Yes	NREM	C3‐A2	Absolute Delta Power	Power Spectral Analysis (FFT; Hanning window tapering) [0.5–3.5 Hz] 128 Hz	No (*t* = −0.23, *p* = 0.82) NS = IN	.	.	No
										Absolute Sigma Power	[12.5–16 Hz]	No (*t* = 1.58, *p* = 0.12) NS = IN	.	.	
(Shao et al. 2022)	46 [IN = 16, NS = 30]	IN: 39 (31,46.3)^h^ NS: 32 (29, 39)^h^	IN: 81 NS: 50	China	Cross‐Sectional	DSM‐5 Insomnia Disorder + ISI score ≥ 15	No	NREM (first half of the night)	Cz‐A1/A2	Spindle Density	Semi‐Automatic Detection (adapted root mean square sigma filtering) [11–16 Hz] (Krystal et al. 2002) 5000 Hz (then down sampled to 500)	Yes (*Z* = −2.15, *p* = 0.032) NS > IN	.	.	No
										Spindle Count		Yes (Z = −2.92, *p* = 0.004) NS > IN	.	.	

										Spindle Duration		No (*Z* = −0.44, *p* = 0.661) NS = IN	.	.	

										Spindle Amplitude		No (t = 1.18, *p* = 0.253) NS = IN	.	.	
(Spiegelhalder et al. 2012)	54 [IN = 25, NS = 29]	IN:47.8 ± 7.2 NS:46.5 ± 5.0	IN: 64 NS: 62	German	Cross‐Sectional	DSM‐IV‐TR Primary Insomnia	No	NREM2	C3‐right earlobe	Absolute SO Power	Power Spectral Analysis (FFT) [0.1–1 Hz] (Press and Rybicki 1989) 200 Hz	No (*t* = −0.81, *p* = 0.421) NS = IN	No (*p* > 0.1) *F* = *M*	.	All females studied during follicular phase
										Absolute Delta Power	[1–3.5 Hz]	No (*t* = 1.46, *p* = 0.150) NS = IN	No (*p* > 0.1) *F* = *M*	.	
										Absolute Sigma Power	[12–16 Hz]	Yes (t = 3.25, *p* = 0.002) NS < IN	No (*p* > 0.1) *F* = *M*	.	
(Svetnik et al. 2017)	1614 [IN = 803 (YA:417 OA:386), NS = 811]	INYA: 44.4 ± 11.1^F^ INYA: 42.8 ± 11.^M^ INOA: 70.7 ± 4.9^F^ INOA: 71.6 ± 5.5^M^ NS: 31.7 ± 10.7^F^ NS: 28.8 ± 8.1^M^	INYA:66 INOA:61 NS:62	USA	Cross‐Sectional	DSM‐IV Primary Insomnia + PSQI score > 5	Yes	NREM	C3‐A2, C4‐A1, O2‐A1, O1‐A2	Absolute Delta Power	Power Spectral Analysis (FFT; hamming window tapering) [1–4 Hz] (Press and Rybicki 1989) Sampling Rate^d^	Yes (*F*_1,145_ = 4.94, *p* = 0.028) NS > IN < 40–45 years	Yes^f^ *F* > *M* becomes larger in older ages	Yes^f^ FIN > FNS > 45y.o. last 2/3 night	No
										Relative Delta Power		No^f^ NS = IN	No^f^ *F* = *M*	.	
										Delta Power Decline		No^f^ NS = IN	No (*p* > 0.05) *F* = *M*	.	
										Absolute Sigma Power	[13–15 Hz]	No^f^ NS = IN	Yes^f^ *F* > *M* becomes larger in older ages	Yes^f^ Larger sex‐based differences in PI than NS	
										Relative Sigma Power		.	No^f^ *F* = *M*	Yes^f^ FIN < FNS < 40–45y.o 1st 1/3 night	
										Sigma Power Decline		No^f^ NS = IN	No (*p* > 0.05) *F* = *M*		
(Wu et al. 2013)	82 [IN = 50, NS = 32]	IN:36.3 ± 8.9 NS:32.7 ± 9.3	IN: 50 NS: 59	USA	Cohort	DSM‐IV Primary Insomnia + PSQI score > 5	Yes	NREM	C3, C4‐A1/A	Absolute Delta Power [0.5–4 Hz]	Power Spectral Analysis (FFT; hamming window tapering) Sampling Rate^d^	No (F_1,62_ = 0.33, *p* = 0.57) NS = IN	No^f^ *F* = *M*	.	No
										Absolute Sigma Power [12–16 Hz]		No (F_1,62_ = 0.04, *p* = 0.83) NS = IN	No^f^ *F* = *M*	.	

*Note:* NREM‐N2 + N3 + N4 (in papers older than 2007).

Abbreviations: *F*, female; *M*, male; OA, older adult; YA, young‐adult.

^a^ No mean or SD provided.

^b^ Unspecified channel reported for statistical analyses.

^c^ No SD provided.

^d^ Unspecified.

^e^ Calculated from data provided.

^f^ No statistics provided.

^g^ Especially prominent.

^h^ Quartiles.

^i^ Bins self‐defined rather than spectral bands.

#### 3.3.3 Insomnia and Normal Sleepers

Most studies (80%) investigating brain oscillations in IN and NS did not examine sex‐based differences (i.e., perform a main effect of sex or group‐by‐sex interaction analyses). Forty‐four papers with both normal sleepers and persons with insomnia were included in the systematic review. Of those, 8 directly compared sex‐based differences in brain oscillations during sleep (see Table 4). Out of the 8 studies evaluating sex‐based difference: 8 reported on spindle characteristics, 6 reported on SWA characteristics, and 1 reported specifically on SO characteristics. The results are shown based on the main differences between the groups (NS vs. IN) and whether these differences depend on sex (i.e., sex by group interaction). When relevant data was provided, the overall effect of sex was also included. Half of the studies (50%) used DSM‐IV criteria for primary insomnia, which define it as difficulty falling or staying asleep for at least 1 month without another underlying cause. A quarter (25%) used chronic insomnia criteria from DSM‐III/IV/V, which require symptoms to persist for at least 3 months. A smaller number of studies (12.5%) focused on sleep maintenance insomnia (characterized by frequent nighttime awakenings; DSM‐III) or paradoxical insomnia, where individuals report poor sleep despite objective evidence of adequate sleep (ICSD‐3). Because so few studies used alternative diagnostic definitions, it's difficult to determine whether the type of classification meaningfully influenced the findings. Overall, findings on sex‐based differences in sleep‐related brain oscillations in individuals with and without insomnia were inconclusive and varied by study method, age, and analysis type.

##### 3.3.3.1 Spindle Activity

Eight research papers explored sex‐based differences in spindle characteristics in normal sleepers and persons with insomnia, representing a total of 2063 participants: 1043 with insomnia (IN; mean age = 53.50 ± 9.20, median group size = 39, range 12 to 417) and 1020 normal sleepers (NS; mean age = 40.90 ± 9.20, median group size = 29.50, range 20 to 811; See Table 4). There were 6 differing spindle characteristic outcomes (spindle density, frequency, amplitude, duration, count, and sigma power). No clear or consistent sex‐based differences were found in spindle characteristics, although some studies showed slight decreases in sigma power, particularly in older females with insomnia, but results were inconsistent.

###### 3.3.3.1.1 Spindle (Event‐Based Detection)

No studies have considered a sex*group interaction in any of the spindle characteristics. When considering the entire sample rather than by group, 1 study (50%; (Benbir Şenel et al. 2021)) reported no significant sex‐based differences on spindle density, duration, and frequency, but did not report statistical data. The other paper on spindle duration, count, and amplitude only compared groups (IN vs. NS) without analysing sex‐based differences (see Table 4) (Shao et al. 2022).

###### 3.3.3.1.2 Sigma Spectral Power

When investigating sex effects, 6 studies examined sex‐based differences in sigma power in NS and IN. Of those, 83% (*k* = 5) (Spiegelhalder et al. 2012; Buysse et al. 2008; Svetnik et al. 2017; Wu et al. 2013; Krystal et al. 2002) found no significant effects in either absolute or relative power, with only 20% providing statistical support (Spiegelhalder et al. 2012; Buysse et al. 2008). Moreover, 2 studies reported a group‐by‐sex interaction. One found that female IN had significantly greater absolute sigma power than female NS, while no significant differences were observed between male IN and male NS, nor were there sex‐based differences within either group (Buysse et al. 2008). The other study reported that although there were no group differences in absolute sigma power, there were larger sex‐based differences within the IN group than NS, and the sex effect was more apparent in older ages (Svetnik et al. 2017). Conversely, they found that female NS had significantly higher relative sigma power than female IN, specifically in those under 40–45 years old during the first third of the night (Svetnik et al. 2017). The remaining article only compared groups without analysing sex‐based differences in sigma power (Lunsford‐Avery et al. 2021). The sole study on sigma power decline across the lifespan (Svetnik et al. 2017) reported no group or sex‐based differences in all parts of the night but did not assess group‐by‐sex effects. This indicates that the rate of absolute sigma power decline with age was similar for both females and males across IN and NS groups and throughout all parts of the night.

##### 3.3.3.2 Slow Wave Activity

Six research papers explored sex‐based differences in SWA characteristics in normal sleepers and individuals with insomnia. The 6 studies reported a total of 1977 participants: 1007 with insomnia (IN; mean age = 42.50 ± 10.10, median group size = 49, range 25 to 803) and 970 normal sleepers (NS; mean age = 40.80 ± 9.50, median group size = 30.5, range 20 to 811; See Table 4) and 2 differing SWA characteristic outcomes (delta and SO power). Studies investigating SWA showed mixed results, with some studies finding higher delta power in females with insomnia, particularly in middle (23–38 years) and older (> 45 years) age groups.

###### 3.3.3.2.1 Slow Wave and Slow Oscillation (Event‐Based Detection)

No studies to date have examined group‐by‐sex‐based differences in SW or SO characteristics using event detection.

###### 3.3.3.2.2 Delta and Slow Oscillation Spectral Power

When investigating sex effects, the most commonly reported domain was delta power (*k* = 6). Of papers on delta spectral power (*k* = 6), 83% (*k* = 5) found no sex‐based differences in relative (Buysse et al. 2008; Svetnik et al. 2017) or absolute (Spiegelhalder et al. 2012; Buysse et al. 2008; Wu et al. 2013; Krystal et al. 2002) delta power, while 1 (14%) found sex‐based differences (Svetnik et al. 2017) that became more apparent in older ages. Specifically, they found females had greater absolute SWA, but no differences in relative delta power. Two studies (29%) investigated group‐by‐sex interaction and both found that female IN had higher absolute delta power compared to female NS (Buysse et al. 2008; Svetnik et al. 2017). However, Svetnik et al. (2017) found this relationship specifically in females older than 45 years and in the last two‐thirds of the night, whereas Buysse et al. (2008) found this for the full NREM period in their middle‐aged female population. A group‐by‐sex interaction was not observed on relative delta power in both of these studies. However, 1 found (Buysse et al. 2008) that male NS had higher relative delta power compared to females (a contrast to the findings in our NS only section). Similarly, 1 paper investigating delta power decline (i.e., across the lifespan) reported no sex‐based differences in all parts of the night (Svetnik et al. 2017), but did not assess group‐by‐sex effects. This indicates that the rate of absolute power decline with age in the delta band was similar for both females and males, and throughout all parts of the night. The only study on SO power (< 1.25 Hz) found no statistically significant differences between sexes and did not examine group‐by‐sex interactions (Spiegelhalder et al. 2012).

### 3.4 Findings From the Meta‐Analysis

While there were enough eligible papers and statistical data for meta‐analysis of sex‐based differences on brain oscillations in normal sleepers, there was insufficient statistical data provided by papers to meta‐analyse the effect of sex on brain oscillations in insomnia compared to normal sleepers.

#### 3.4.1 Overall Effect of Sex on Spindles in Normal Sleepers

Two (Gaillard and Blois 1981; Ujma et al. 2014) of the 8 studies on spindle density in normal sleepers were eligible for meta‐analysis, revealing a moderate sex‐based difference effect, favouring females (*n* = 190, SMD −0.65 [95% CI: −1.95, 0.66], *I* ^2^ = 85%—Figure 2A). Of the 8 studies on absolute and normalized sigma power in NS, 5 provided enough data to allow meta‐analysis of absolute sigma power (Hertenstein et al. 2018; Richards et al. 2013; Plante et al. 2013; Ehlers and Kupfer 1997; Buysse et al. 2008), resulting in a statistically significant but small effect size (*n* = 365, SMD −0.29 [95% CI: −0.50, −0.08], *I* ^2^ = 0%—Figure 2B) favouring females. There were not enough papers reporting data for meta‐analysis on the effects of sex on other spindle characteristics (e.g., relative sigma power, spindle density and duration).

**FIGURE 2:**
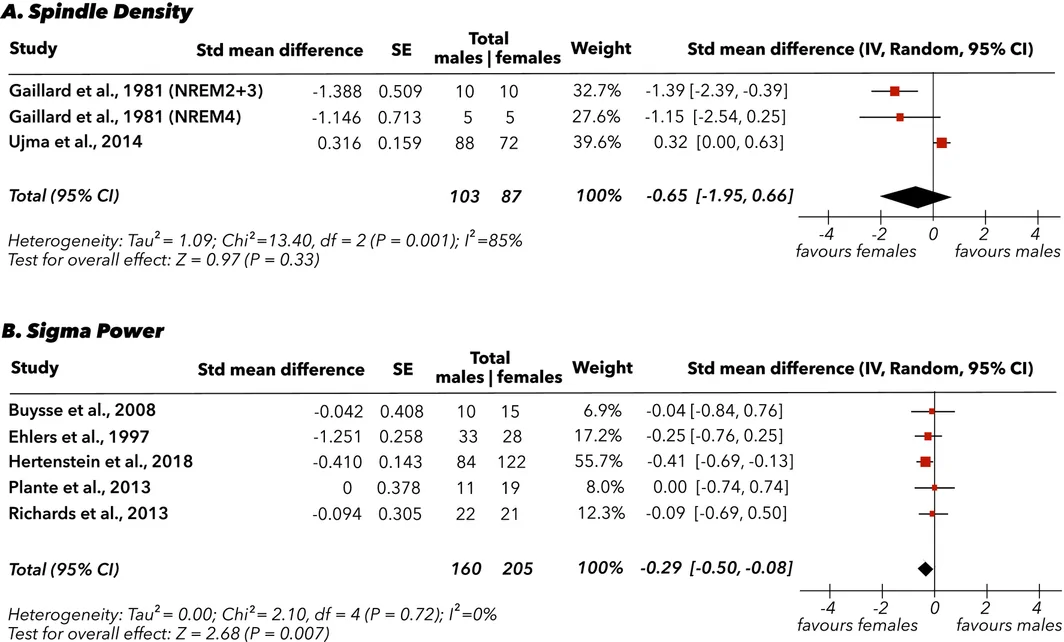
Synthesis and forest plot for spindle outcomes [(A) spindle density; (B) sigma power] of male vs. female. CI, confidence interval; SE, standard error; Std., standardized.

#### 3.4.2 Overall Effect of Sex on Slow Wave Activity in Normal Sleepers

Seven of the 19 studies on delta power were eligible for meta‐analysis. The analysis of 6 studies (Figure 3) on absolute and 1 on relative delta power in normal sleepers showed a statistically significant difference, favouring females with a moderate effect size (*n* = 407, SMD −0.50 [95% CI: −0.70, −0.30], *I* ^2^ = 0%). There was insufficient data provided by papers to meta‐analyse the effect of sex on SW and SO (based on event detection) characteristics.

**FIGURE 3:**
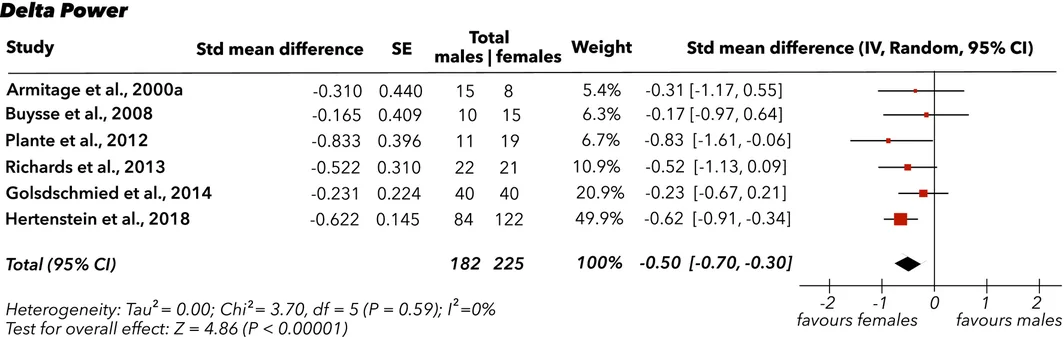
Synthesis and forest plot for delta power outcomes of male vs. female. CI, confidence interval; SE, standard error; Std., standardized.

### 3.5 Heterogeneity

Heterogeneity across studies was observed, particularly in the reporting of spindle and SWA characteristics. Significant heterogeneity was found across studies for sleep spindle density (*I* ^2^ = 85%), but there was no observed heterogeneity among sigma power (*I* ^2^ = 0%) or delta power (*I* ^2^ = 0%).

Sensitivity analyses were conducted to assess heterogeneity and the robustness of findings. Leave‐one‐out analyses indicated that no single study disproportionately influenced the results. Publication bias and small‐study effects were evaluated using funnel plots of effect size (Hedges' *g*) by standard error, which showed no evidence of asymmetry, suggesting no substantial reporting or publication bias across outcomes (i.e., sleep spindle density, sigma power, and SWA).

## 4 Discussion

To the best of our knowledge, this is the first systematic review and meta‐analysis to examine the effects of biological sex on sleep spindles and SWA in adults with and without insomnia and to directly assess whether insomnia modifies these sex‐related patterns in quantitative EEG measures (e.g., event‐based detection, power spectral analysis). We found small to moderate effects for higher spindle density, sigma power, and delta power among females compared to male normal sleepers. Our review also highlights critical gaps in the literature for sex‐based differences in brain oscillations during sleep.

### 4.1 Sex‐Based Differences in Spindle and Slow Wave Activity in Normal Sleepers

Sleep spindles and SWA are key oscillatory features of NREM sleep, involved in synaptic plasticity, memory consolidation, and cognitive regulation (Timofeev and Chauvette 2017; Dang‐Vu et al. 2008, 2010; Fernandez and Lüthi 2020). Evidence from the systematic review and our meta‐analysis found modest but consistent sex‐based differences in both spindle and SWA characteristics, generally favouring females. Few studies found sex‐based differences in spindle density (Gaillard and Blois 1981; Ujma et al. 2014), but those that did typically reported higher values in females (Djonlagic et al. 2021; Ujma et al. 2014). Our results confirmed a moderate female advantage in spindle density, most rated the quality as good to satisfactory. All 5 papers that failed to detect sex differences had small sample sizes, likely a result of insufficient statistical power, analysed spindles only during NREM2 (Huupponen et al. 2002; Santamaria et al. 2000; Bové et al. 1994; Ritter et al. 2018; Gorgoni et al. 2016), and had inconsistent methods and protocols (e.g., EEG channels, sigma frequency definitions, sampling rates, participant age, use of an adaptation night; see Table 2). Although the evidence for other spindle characteristics (e.g., amplitude, duration, and distribution) was more variable and not suitable for quantitative analyses, the overall pattern suggests a modest female advantage in several spindle‐related features. This aligns with findings from the influential study by Purcell et al. (2017), which was excluded from this review due to inclusion of children and persons with sleep apnea in a pooled dataset.

Most studies reported higher absolute and relative sigma power in females (Hertenstein et al. 2018; Djonlagic et al. 2021; Carrier et al. 2001; Dijk et al. 1989; Luca et al. 2015; Ondze et al. 2003), particularly in the during NREM and in central channels Our results confirmed a small but consistent female advantage in absolute sigma power. Those that failed to find significant sex‐based differences in sigma power (Plante et al. 2015; Ondze et al. 2003; Richards et al. 2013; Ehlers and Kupfer 1997) may be due to insufficient statistical power due to limited sample sizes. While relative sigma power could not be included in the meta‐analysis due to an insufficient number of studies, Cave and Barry (2021) reported moderate certainty for no sex‐based differences in middle to older adults. This contrasts with findings from our systematic review indicating that females display greater relative spindle power compared to males (Djonlagic et al. 2021; Luca et al. 2015). This may be due to their inclusion of papers using non‐gold‐standard methods (e.g., at home PSG).

For SWA, most studies examined delta power (0.5–4 Hz), with fewer focusing specifically on SW (< 4 Hz) or SO (< 1.25 Hz) as discrete events. Our systematic review and meta‐analysis found consistent sex differences in delta power, with females generally showing higher absolute values (Hertenstein et al. 2018; Djonlagic et al. 2021; Carrier et al. 2001; Dijk et al. 1989; Latta et al. 2005; Latreille et al. 2011; Munch et al. 2007; Armitage 1995; Colrain et al. 2009; Fukuda et al. 1999; Massicotte‐Marquez et al. 2005; Jones et al. 2024). Some studies highlighted regional effects in parietal and occipital areas (De Gennaro et al. 2017). These studies were mostly of good or satisfactory quality, and the results across studies were very consistent. Studies reporting no sex differences (Luca et al. 2015; Ondze et al. 2003; Richards et al. 2013; Plante et al. 2013, 2012; Goldschmied et al. 2014; Latreille et al. 2011) in absolute delta power often had small samples, older participants, and lacked an adaptation night. In contrast, studies of relative delta power reported no sex‐based differences (Latta et al. 2005; Landolt and Gillin 2005; Munch et al. 2007; Plante et al. 2012; Armitage 1995). This divergence between absolute and relative power likely reflects methodological and anatomical influences rather than genuine neurophysiological sex differences in delta power. Absolute EEG power is strongly affected by factors such as skull thickness and head tissue conductivity, which impact EEG amplitude and differ on average between males and females (Dijk et al. 1989; Rosinvil et al. 2021; Cave and Barry 2021; Panov 2023). By scaling power values within each individual, relative metrics reduce the influence of skull and tissue differences and improve comparability across individuals. The absence of sex effects in relative delta power, therefore, suggests that the differences observed in absolute power may be driven more by global amplitude differences (i.e., all power bands) than by frequency‐specific neural processes. This has been supported previously by Chapman et al.'s (2025) review (Chapman et al. 2025). One study found greater normalized delta power in males (Latta et al. 2005), likely due to atypical normalization procedures. Support for sex‐based differences in event‐based findings on SW and SO features were too inconsistent and heterogeneous for quantitative synthesis, though some studies reported greater SW amplitude in females (Armitage, Hoffmann, Trivedi, and Rush 2000; Carrier et al. 2011; Rosinvil et al. 2021).

Our findings on SWA and spindle characteristics suggest potential sex‐related differences in thalamocortical connectivity, which plays a crucial role in regulating cognitive functions (Fernandez and Lüthi 2020), emotions and has implications on neuroprotection (Neylan and Walsh 2022). Across studies, we observed a stronger convergence in findings on spectral power (i.e., sigma and delta power) compared to event characteristics (i.e., SW and spindle events). This may reflect methodological consistency of spectral analysis, which is more sensitive to sampling variability and detection algorithms.

### 4.2 The Effects of Insomnia on Sex‐Based Differences in Spindle and Slow Wave Activity

There was notable heterogeneity in how insomnia was defined across studies, which may influence the interpretation of results. The findings presented mainly represent the DSM‐IV criteria for primary insomnia (requiring at least 1 month of sleep difficulty [50%]); others employed more stringent definitions, such as DSM‐III/IV/V criteria for chronic insomnia (25%), which require symptoms to persist for at least 3 months. A small subset (12.5%) focused on specific subtypes, such as sleep maintenance insomnia or paradoxical insomnia. Given the limited number of studies using these alternative classifications, it remains unclear whether differences in diagnostic criteria significantly impacted the findings. This is particularly relevant given that the most up‐to‐date diagnostic criteria of insomnia disorder from the DSM‐5 and ICSD‐3 do not distinguish whether insomnia is due to the presence of comorbidities or not (formerly called primary insomnia (American Psychiatric Association 1998)).

#### 4.2.1 Group Differences

We found inconsistent findings for whether spindle activity remains stable regardless of insomnia. A small number of studies reported spindle density is more sensitive to the presence of IN (Benbir Şenel et al. 2021; Shao et al. 2022), supporting existing evidence that reduced spindle density is a reliable marker of disrupted sleep (Fernandez and Lüthi 2020; Chen et al. 2023; Cox et al. 2012). Contrastingly, 2 studies found higher sigma power in IN compared to NS (Spiegelhalder et al. 2012; Krystal et al. 2002), while most found no differences in sigma power, spindle duration, frequency, and amplitude. While spectral measures like sigma and delta power offer stable, trait‐like insights into sleep physiology in normal sleep, they may also miss subtle disruptions seen in insomnia and could explain findings of altered spindle density in insomnia despite inconsistent spectral findings. In contrast, event‐based metrics such as spindle density may better detect state‐dependent disturbances in thalamocortical and cortical function (Dang‐Vu et al. 2010). However, the inconsistent study quality and risk of bias may have influenced these findings (Spiegelhalder et al. 2012; Krystal et al. 2002).

To the best of our knowledge, no studies investigating sex‐based differences have also examined differences between insomnia and normal sleepers in event‐based SW or SO characteristics. Our systematic review also found limited but generally consistent evidence regarding SWA in insomnia. Specifically, the majority of studies found no significant differences between IN and NS in either absolute (Spiegelhalder et al. 2012; Buysse et al. 2008; Wu et al. 2013) or relative (Buysse et al. 2008; Lunsford‐Avery et al. 2021) delta or SO power (< 1.25 Hz) (Spiegelhalder et al. 2012). Our findings contrast with other studies (Huang et al. 2025; Hogan et al. 2020) and systematic reviews/meta‐analysis (Zhao et al. 2021) that report lower absolute and relative delta and SO power in persons with insomnia compared to normal sleepers. This may be due to differences in methods and poorer quality of the studies (e.g., limited generalizability, potential bias from small or unrepresentative samples and non‐responders, inadequate control for confounders, and questionable statistical validity) investigated at both group and sex differences. This might also underscore the heterogeneous nature of insomnia (Kao et al. 2021). Subjective complaints may not align with objective measures (reflecting sleep state misperception (American Academy of Sleep Medicine 2005; Rezaie et al. 2018)) while others may show altered sleep homeostasis (e.g., short sleep duration subtype (Vgontzas et al. 2013)). However, most studies lack detailed reporting on insomnia duration (i.e., chronicity of symptoms) or subtype, which might have more profound impacts on neurophysiology. Emerging evidence also suggests that EEG profiles vary across subtypes. For instance, 1 study found that persons with subjective IN exhibited significantly higher sigma power compared to those with objective IN and NS (Krystal et al. 2002).

#### 4.2.2 Sex‐Based Differences

Six studies tested sex‐based differences in absolute and relative sigma power in IN (Spiegelhalder et al. 2012; Buysse et al. 2008; Lunsford‐Avery et al. 2021; Svetnik et al. 2017; Wu et al. 2013; Krystal et al. 2002). Both studies on relative sigma power found no sex differences. However, 5 reported no sex‐based differences in absolute sigma power (Spiegelhalder et al. 2012; Buysse et al. 2008; Lunsford‐Avery et al. 2021; Wu et al. 2013; Krystal et al. 2002), 1 reported higher absolute sigma power in females when combining IN and NS groups (Svetnik et al. 2017), while the other found no sex‐based difference in the IN group alone (Bader et al. 2013). Only 2 studies tested for sex‐by‐group interactions. One (Buysse et al. 2008) found that middle‐aged female IN exhibited greater absolute sigma power than NS, while the other reported greater absolute sigma power in females than males, with sex differences more pronounced in the IN group than in NS (Svetnik et al. 2017). The opposite pattern was found for relative sigma power (Svetnik et al. 2017)—lower in female IN, specifically during the first third of the night. These discrepancies likely arise from differences in methods—such as using absolute versus relative power, electrode placement (central vs. lateral), insomnia subtypes, and time‐of‐night studied. Studies that found no sex effects in sigma power (Spiegelhalder et al. 2012; Wu et al. 2013; Krystal et al. 2002) or other spindle characteristics (Benbir Şenel et al. 2021; Shao et al. 2022), often combined insomnia and normal sleeper groups or did not test sex or group‐by‐sex interactions systematically. Compared to those that did find significant sex effects, some of these had lower quality, limiting the reliability of their findings (Spiegelhalder et al. 2012; Krystal et al. 2002).

Research on sex‐based differences in SWA and SO features in insomnia is even more sparse and inconclusive, with no studies, to the best of our knowledge, systematically examining sex differences or sex‐by‐group interactions using event detection methods. Of the few that examined SWA in insomnia, only 1 study identified sex‐based differences in absolute delta power (Svetnik et al. 2017), with a female advantage that became more pronounced with age. Every other study reviewed found no significant sex effects in either relative (Bader et al. 2013; Buysse et al. 2008; Svetnik et al. 2017) or absolute (Spiegelhalder et al. 2012; Bader et al. 2013; Hall et al. 2000; Buysse et al. 2008; Wu et al. 2013; Krystal et al. 2002) delta power. Compared to the 1 study finding significance, most of these studies were of unsatisfactory quality, limiting the reliability of their findings and making it difficult to draw firm conclusions (Spiegelhalder et al. 2012; Wu et al. 2013; Krystal et al. 2002). Only 2 studies investigated group‐by‐sex interactions in delta power; both found that female IN had higher absolute delta power compared to female NS (Buysse et al. 2008; Svetnik et al. 2017), 1 specifically noting this in older female IN (> 45 years). However, 1 (Buysse et al. 2008) reported higher relative delta power in male NS compared to females—a finding that contrasts with other studies showing no sex differences in normal sleepers. This discrepancy may reflect population differences (e.g., age, hormonal status), methodological factors, or an artificial inflation from using relative power. In this case, higher relative delta power in males may not reflect stronger SWA, but rather lower overall EEG power compared to females, making delta proportionally larger.

Given the quality concerns (i.e., high risk of bias, small samples, and methodological inconsistencies), and heterogeneity in insomnia criteria, it remains difficult to conclude whether sex‐based differences are present in NREM oscillatory activity in insomnia disorder. Systematic reviews reinforce these findings, noting that while sex‐based differences in spindle and SWA are robust in normal sleeping populations (i.e., mostly favouring females), they are not consistently replicated in clinical insomnia samples (Weiner and Dang‐Vu 2016).

### 4.3 Unravelling the Female Insomnia Paradox

Females generally exhibit higher spindle density, SWA, and longer total sleep time—features typically associated with deeper, more restorative sleep. Yet, they are nearly twice as likely as males to develop insomnia disorder (Morin and Jarrin 2022). Although healthy females typically show favourable sleep features, it is unclear whether these are preserved in females with insomnia, as few studies have examined group‐by‐sex interactions and female‐specific group analyses. These physiological advantages may be diminished in insomnia or may not fully protect against stressors and stress reactivity more prevalent in females—such as hormonal fluctuations (e.g., menopause), caregiving demands and may further heighten vulnerability to sleep disruption (Kalmbach et al. 2018). Additionally, standard EEG measures may not capture subjective sleep experiences or arousal regulation, both of which contribute to insomnia. Thus, this paradox underscores the need to examine sex‐specific sleep physiology across normal and clinical sleep, integrating biological rhythms with psychosocial context and lived experience.

### 4.4 Layers of Influence: Age as the Thread Connecting Sex, Hormones, and Insomnia

#### 4.4.1 Insomnia

Insomnia prevalence and manifestation are highly dependent on both age and sex given the role of hormonal changes across the lifespan. Indeed, sex disparities in sleep only appear at puberty, and the risk for developing insomnia increases nearly threefold after menarche, and continues to spike during key reproductive transitions, such as pregnancy, peri‐ and post‐menopause (Johnson et al. 2006; Krishnan and Collop 2006; Lee and Kryger 2002). During the menopausal transition and reduction in ovarian hormones, insomnia prevalence rises from 35% to 53% (Krishnan and Collop 2006; Yaffe et al. 1998). Conversely, menopausal hormone therapy (MHT; i.e., bioidentical or low‐dose oestrogens and progestogens) has been shown to improve sleep quality in perimenopausal females (Lord et al. 2014; Moline et al. 2004; Davis and Baber 2022), while hormonal contraceptives (i.e., synthetic ethinyl estradiol and progestins) report more severe insomnia symptoms than non‐users (Bezerra et al. 2020). Changes in male gonadal steroid levels across the lifespan (e.g., andropause, anabolic androgenic steroids use), are also associated with poorer sleep quantity and quality (Lord et al. 2014; Barrett‐Connor et al. 2008; Venâncio et al. 2008). These findings underscore age as a central thread‐ shaping hormonal transitions, modulating the brain's sleep‐related oscillatory activity, and showing a consistent, linear relationship with sleep complaints. In this way, age exerts a powerful and consistent influence shaping, amplifying, or masking the effects of sex‐based differences on sleep.

#### 4.4.2 Age

Age is a critical factor that underlies variability in sex‐based differences in spindles and SWA across normal and insomnia populations. Both spindle characteristics and SWA decline significantly with age. It has been shown that spindle activity peaks in early adulthood and diminishes with aging due to structural and functional brain changes. This includes normal neuronal loss, reduced thalamocortical connectivity, diminished GABAergic neurotransmission, and cortical thinning (Purcell et al. 2017; Fogel and Smith 2011; Mander et al. 2017). Similarly, delta power declines gradually throughout adulthood, reflecting neurobiological deterioration—particularly in the frontal cortex—rather than altered sleep patterns alone. These age‐related changes reduce the brain's capacity for deep, restorative sleep, leading to shallower, fragmented sleep and contributing to the increased prevalence of insomnia in older adults (Mander et al. 2017; Varga et al. 2016). Spindle and SWA are also linked to cognitive health, raising the stakes for understanding their modulation by age and insomnia. For instance, reduced spindle and SWA are associated with cognitive impairment and increased risk for Alzheimer's disease (AD) (Páez et al. 2025; Ujma 2021). Since females represent approximately two‐thirds of AD cases, with oestrogen decline exacerbating this trend, understanding how age and sex interact to shape sleep oscillations is key to both sleep health and cognitive long‐term cognitive trajectories. Insomnia emerging in midlife is also linked to subjective cognitive decline, a potential early sign of mild cognitive impairment (and possible dementia), and associated with objective memory decline in males (Cross et al. 2019; Zhao et al. 2022). Few studies in this review directly examined age‐by‐sex interactions; however, some evidence suggests that differences in spindle and SWA between individuals with insomnia and normal sleepers may vary across the lifespan. For example, Svetnik et al. (2017) (Svetnik et al. 2017) found significant group‐by‐age effects in females only: younger and middle‐aged females with insomnia (< 40 years) showed lower relative sigma power compared to normal sleepers, while older females with insomnia (> 45 years) showed greater absolute delta power than age‐matched controls. These findings suggest that the impact of insomnia on sleep microarchitecture may evolve with age in females, potentially reflecting age‐related neurobiological changes. Given the wide age range in our review (23.6–77.1 years) and the limited number of eligible studies, age‐stratified analyses were not feasible, complicating interpretation of age‐related effects.

#### 4.4.3 Sex Hormones

Hormonal changes across the lifespan likely shape sex‐specific patterns in sleep physiology. Sex‐steroid hormones (i.e., oestrogen, progesterone, and androgens), while primarily are important for maintaining hormonal homeostasis, also play complementary roles in supporting sleep by influencing brain regions involved in sleep regulation (e.g., modulating thalamocortical oscillatory activity). This can be observed in rodent studies which demonstrate that removing gonadal steroids eliminates sex‐based differences in sleep architecture (Paul et al. 2009; Schwierin et al. 1998). Estradiol and progesterone help quiet brain activity by enhancing GABAergic inhibition in areas that generate sleep spindles (i.e., thalamic reticular nuclei and cortical layers), making these rhythms more likely to occur (Baker et al. 2019; Genzel et al. 2012). For example, spindle density increases during the luteal phase (high estradiol and progesterone) and with oral contraceptive use (Plamberger et al. 2021), while combined hormone replacement therapy (HRT) or single‐hormone regimens are associated with higher spindle activity in pre and postmenopausal females (Baker et al. 2019; Antonijevic et al. 2000; Plante and Goldstein 2013). Testosterone may also modulate spindle activity, but the evidence is mixed and less consistent. Animal studies show that gonadal steroid injections in castrated males and ovariectomized females eliminate sex‐based differences in sleep spindle expression implying an interaction between androgens and underlying sex‐based neural circuitry (Mong et al. 2011). In humans, testosterone fluctuations (such as in aging men or women with polycystic ovary syndrome) may alter spindle characteristics given the impacts on poor sleep quality, but this remains understudied (Hachul et al. 2019).

While SWA appears less sensitive to hormonal fluctuations, likely due to its regulation by sleep homeostasis, some evidence suggests hormones still play a modulatory role. In animal models, estradiol suppresses delta power by acting on sleep–wake regulatory regions (e.g., basal forebrain and hypothalamus) (Schwierin et al. 1998; Mong et al. 2011; Smith and Mong 2019), yet human studies show little change in SWA during peri‐menopause despite steep declines in estradiol (Baker et al. 2015). This may possibly be due to gradual hormonal changes and compensatory neuroadaptation that may preserve sleep homeostasis despite estradiol fluctuations (Mong et al. 2011; Smith and Mong 2019). It has been hypothesized that progesterone could enhance deep sleep, however progesterone's effects on SWA are inconsistent. Some studies report increases in delta power when progesterone is naturally high and others show reductions or no change (Brown and Gervais 2020; De Zambotti et al. 2015). Androgens on the other hand may influence SWA more directly by regulating sleep homeostasis. Testosterone acts on androgen receptors in cortical and subcortical structures, with particular influence on the frontal cortex—a key region for SWA generation (Tobiansky et al. 2018). In animal studies, testosterone depletion (e.g., gonadectomy) reduces SWA, while testosterone replacement restores it (Paul et al. 2009). In humans, however, higher testosterone levels in males either endogenous or via therapy (e.g., in males with congenital GnRH deficiency) are linked to reduced SW sleep and delta power in males, even after controlling for age and other covariates (Mong et al. 2011; Ismailogullari et al. 2011). Findings in females remain inconsistent (Hernández‐Pérez et al. 2024), and andropause and SWA have not been directly studied. Overall, estradiol and progesterone enhance spindles via GABAergic mechanisms, but their effects on SWA are mixed or neutral. Testosterone may influence cortical structures involved in SWA, but it's effects on spindle and SWA remain understudied.

Hormonal fluctuations seem to directly and indirectly effect sleep quality. Yet, the majority of studies (34 studies reviewed in this paper [80%] do not consider key hormonal variables that can modulate EEG activity. Of the 42 studies reviewed, only 8 explicitly controlled for hormonal factors affecting sleep or EEG (Bové et al. 1994; Dijk et al. 1989; Richards et al. 2013; Ehlers and Kupfer 1997; Carrier et al. 2011; Armitage, Hoffmann, Fitch, et al. 2000; Latta et al. 2005; Spiegelhalder et al. 2012)). These controls included excluding hormonal contraceptive or HRT users, selecting based on menopausal status or FSH levels, and timing data collection to specific menstrual phases (e.g., follicular phase). Some also ensured participants were not menstruating or reported contraceptive use.

### 4.5 Lack of Sex‐Based Research in Sleep Brain Oscillations

Despite growing recognition of sex as a key biological variable, our review demonstrates that sex‐based differences in spindle and SWA characteristics are infrequently and inconsistently examined in sleep research. While some accounted for sex through design elements like matched controls or covariates, few examined it directly (See Supporting Information). Even of the sex‐based difference studies, sex was a secondary variable of interest. While most studies met basic methodological standards, only 5% were high quality, and nearly one‐fifth were unsatisfactory. Common issues included small, sex‐imbalanced samples, lumping data from both groups, lack of statistical support for sex effects, and failure to test sex‐by‐group interactions—which, according to simulation studies, can reduce detection power of sex‐specific effects by up to 28% and reduce reliability (Mannino et al. 2024).

### 4.6 Strengths, Limitations and Future Direction

Our systematic review and meta‐analysis have several strengths. We used comprehensive search strategies without language or date limits, addressing a key limitation of prior reviews. By examining sex differences in sleep spindles and SWA in both normal sleepers and persons with insomnia, we offer a broader view of how biological sex influences brain rhythms during sleep (See Figure 4). Uniquely, we also explored how sex‐steroid hormone fluctuations interact with thalamocortical synchronization to shape insomnia vulnerability. This mechanistic perspective not only clarifies sex differences in sleep disorders but also points to potential biomarkers for personalized treatments—such as targeting spindle deficits in stress‐prone females or restoring SWA in perimenopausal women. By connecting neural activity, hormonal changes, and subjective sleep experience, our review advances a more biologically grounded understanding of sex‐specific insomnia and highlights key gaps in the current evidence base.

**FIGURE 4:**
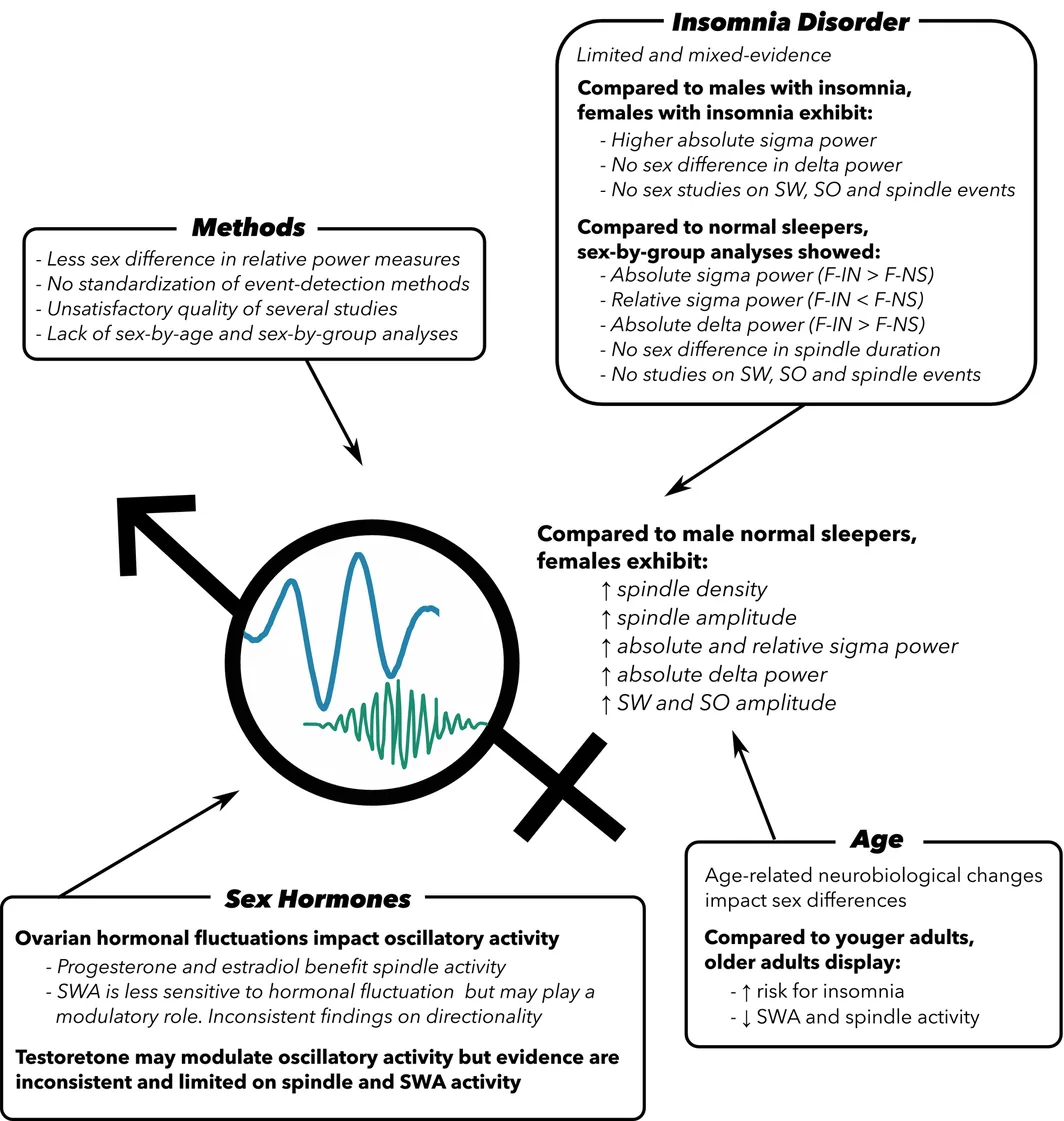
Overview of research on participant and study design factors affecting sleep oscillations.

However, our systematic review also had limitations. The limited number of available studies, considerable variability in study methods, incomplete data (e.g., missing effect estimates or sample characteristics), and lack of information on the chronicity of insomnia limit the scope of some of our findings and inhibit analysis depth. Additionally, methodological quality was moderate but inconsistent, and insomnia studies were of lower quality, highlighting a major limitation of the body of evidence in brain oscillations during sleep. Sufficiently well‐powered, sex‐stratified analyses are needed to clarify sex effects on NREM oscillations, particularly in insomnia. Greater standardization of study and brain‐oscillation analysis methods would improve the reliability, consistency, and comparability of findings across studies. To offer a more comprehensive and sex‐sensitive approach, we recommend studies report both absolute and relative power and include event‐based measures.

Sex differences are rarely prioritized, often examined in underpowered secondary analyses with unbalanced samples, and group‐by‐sex interactions are seldom tested. Despite longstanding calls for more sex‐balanced samples, this continues to be an area of further attention and development in sleep research. Historically, females were excluded from sleep research due to hormonal variability, resulting in a male‐biased evidence base (Liu et al. 2003). Though both sexes are now included, many studies still overlook hormonal influences which are important for improving our understanding of sleep neurophysiology and insomnia risk. This shift from exclusion to unrestricted inclusion may limit progress to better characterize and understand sleep. This can be circumscribed by sex‐informed, hormonally aware methods, using adequately powered, stratified designs to clarify how sex, age, and insomnia interact to shape sleep neurophysiology.

Our systematic review primarily focuses on biological sex rather than gender (i.e., a societal construct) and gender identity (i.e., a personal construct), all of which can significantly impact sleep (Harry‐Hernandez et al. 2020; Mallampalli and Carter 2014). Gender‐related stressors and discrimination contribute to higher insomnia rates in women and non‐cisgender persons, yet neurophysiological studies in these groups are scarce (Caceres et al. 2022). There is a need for sex inclusive (e.g., how androgens shape sleep dynamics across the lifespan) and gender‐inclusive biopsychosocial investigations in sleep science.

## 5 Conclusion

Sex, hormones, and age each play a pivotal role in shaping sleep neurophysiology, yet they remain underexamined in brain‐oscillations sleep research, especially in the context of insomnia. Research in this area is increasingly important, as highlighted by Graham and colleagues “Statement on the Importance of Sexuality and Gender Research” (Graham et al. 2025) released in 2025 which emphasizes the need to understand how these factors intersect with health, behaviour, and social well‐being. With evolving societal attitudes and growing recognition of gender diversity, it is increasingly essential to expand research in this field. Future research can adopt sex‐and gender‐informed methods, control for hormonal status, and stratify by age. This can clarify whether true sex‐based differences exist in insomnia‐related oscillatory activity and support more precise, personalized insomnia interventions that address health disparities. Such work can play a crucial role in advancing health outcomes and ensuring that clinical interventions are inclusive and tailored to the diverse needs of all persons, ultimately helping to enhance sleep and cognitive outcomes across the lifespan.

## Author Contributions

**Nyissa A. Walsh:** conceptualization, data curation, formal analysis, visualization, writing – original draft. **Aurore A. Perrault:** conceptualization, visualization, supervision, writing – original draft. **Emma‐Maria Phillips:** data curation, writing – review and editing. **Arsenio Páez:** visualization, formal analysis, writing – review and editing. **Thien Thanh Dang‐Vu:** supervision, writing – review and editing. **Nathan E. Cross:** writing – review and editing.

## Funding

N.A.W. received financial support from the Social Sciences and Humanities Research Council and local scholarships (Concordia University) (767‐2023‐2413). All other authors received funding not related to this project.

## Conflicts of Interest

T.D.V. has received consultant and speaker fees from Eisai, Idorsia, Takeda, and Paladin Labs, and grants from Jazz Pharmaceuticals and Paladin Labs. All other authors declare that they have no relevant financial interests or personal relationships that could have influenced the findings presented in this paper.

## Supporting information


**Figure S1:** Search strategy.

**Table A1:** Glossary of terms.

## Data Availability

The data that support the findings of this study are available from the corresponding author upon reasonable request.
