# Supplementary material for: A Systematic Review and Meta‐Analysis of Biological Sex Differences in Sleep Spindles and Slow Wave Activity in Adults With and Without Insomnia
Source: J Sleep Res. 2026 Jun 22;35(5):e70388. doi: 10.1111/jsr.70388 (PMC13619051; doi:10.1111/jsr.70388)
Supplement: Supplementary file 1 [file JSR-35-e70388-s001.docx]

**Supplementary Material**

***Figure S1: Search Strategy***

***Underrepresentation of Sex-Based Analyses in Insomnia Research***

In the original search, 32 studies were excluded as they focused only on one sex. Of the studies retained (n= 257), 83% matched the criteria for population and outcomes but did not analyse sex difference (n= 214). Among the eligible studies 14 different countries considered sex differences in brain oscillations in NS and/or individuals with IN. Of the 214 studies that match criteria for population and outcomes but did not analyses sex difference per se, 97 did not list a reason, 64 adjusted for sex (7 articles used sex as a covariate, 8 collected similar sex distribution controls, 47 collected sex-matched controls [2 of which acknowledged the differences by sex in the introduction], 1 paper had all participants in the same menstrual phase, and 1 paper had all females on contraceptives), 5 compared sex for different variables (e.g., %NREM2) but not brain oscillations, and 10 papers acknowledged the impact of sex on brain oscillations in the discussion (7 articles listed not considering sex differences as a limitation, acknowledging the potential impact of female cortisol hormonal fluctuations on brain oscillations [e.g., menstrual cycle phase, menopause, and hormonal contraceptive use], 2 indicated their study was insufficiently powered, and 1 indicated it was outside the scope of the paper).

***Attrition and Acknowledgement of Sex-based Difference per Population Study (NS, IN, NS & IN)***

*Normal Sleepers*

Of the 183 studies on NS that met our eligibility criteria, 33 (18%) directly examined sex-based differences in brain oscillations during sleep, while 150 did not. Of these 150 studies, 56 adjusted for sex in their analyses: 49 used age- and sex-matched controls, 1 further matched participants by menstrual cycle phase, and 6 included sex as a covariate. Among the remaining 94 studies, only 4 provided reasons for not comparing sex-based differences: 2 cited insufficient statistical power, 1 found no significant differences and did not consider sex a confounder, and 1 deemed it outside the study's scope but discussed evidence of sex-based differences in their discussion. The remaining 92 studies did not explain their omission, though 1 noted that gender discrepancies might explain differences in results compared to other studies.

*Insomnia*

Based on our eligibility criteria, 17 studies on individuals with IN were deemed suitable. Of those 17 papers, 2 (12%) directly compared sex-based differences in brain oscillations during sleep, while 15 studies did not consider sex-based differences. One of these 15 studies adjusted for sex in their analyses, although they did not list whether there were sex-based differences in sleep spindles in those with IN. 0/14 of the other studies listed reasons why they did not compare sex-based differences.

*Insomnia and Normal Sleepers*

Lastly, we systematically reviewed papers that contained both NS and those with IN. Based on our eligibility criteria, 41 studies with both NS and those with IN were deemed suitable. Of those 41 papers, 8 (20%) directly compared sex-based differences in brain oscillations during sleep (see Table 3), while 36 studies did not. Of these 36 studies, 16 adjusted for sex in their analyses: 9 used age- and sex-matched controls and 7 included sex as a covariate. Eleven of the 36 studied simply indicated that sex distribution was similar between IN and normal sleeper groups. Among the remaining 9 studies, only 2 provided reasons for not comparing sex-based differences, both citing insufficient statistical power but discussed evidence of sex-based differences in their discussion. The remaining 7 studies did not explain their omission.

**RESULTS**

***Group Differences in Neural Oscillations in Studies on*** ***Insomnia and Normal Sleepers***

*Spindle (event-based detection)-* When investigating group effects without considering sex-based differences, the most reported domains were spindle density and duration (k = 2). Spindle detection method varied between papers (see **Table 4**). Neither studies reported any difference in spindle duration between NS and IN[101,102], even when further dividing spindles into slow or fast spindle activity (using Cz in the 11–16 Hz range and an unspecified range in C3/F4, respectively). The same studies found that NS had significantly higher spindle count[102] and density (combined, fast, and slow[101,102]) compared to IN. No group differences were found in spindle frequency[101] and amplitude[102].

*Sigma Spectral Power-* When investigating group effects without considering sex-based differences, sigma power was the most commonly reported domain in power spectral analysis (k = 7). Of the studies examining group differences in sigma power in NS and IN, 57% (k= 4)[103–106] found no significant differences between groups for absolute and relative power. Two studies (29%)[96,107] found that IN had greater absolute and relative sigma power compared to NS, with one of the studies finding this to be true for both subjective and objective insomnia[107]. Specifically, those with subjective insomnia (previously called paradoxical insomnia-[108]) had significantly higher sigma power than objective insomnia (previously categorized as psychophysiological insomnia) and NS[107]. The sole study on sigma power decline across the lifespan[105] reported no group differences in all parts of the night. This indicates that the rate of absolute sigma power decline with age was similar for both IN and NS groups, and throughout all parts of the night.

*Insomnia and Normal Sleepers: Slow Wave Activity*

*Slow Wave and Slow Oscillation (event-based detection).* No studies to date have examined group-based differences in SW or SO characteristics using event detection.

*Delta and Slow Oscillation Spectral Power*- When investigating group effects without considering sex-based differences, the most commonly reported domain was delta power (k= 6). Of papers on delta spectral power, 67% (k= 4) found no group differences in relative[103] or absolute[96,103,104,106] delta power, while 29% (k= 2) found a group difference—one for relative but not absolute[107], and the other for absolute but not relative[105]. One study (14%) found that IN had higher absolute SWA compared to NS, specifically in those under 40–45 years old during the first third of the night[105]. The other (14%) found the opposite, where NS had greater relative SWA compared to subjective IN, but not objective IN[107]. Similarly, one paper investigating SW power decline (i.e., across the lifespan) reported no group differences in all parts of the night[105]. This indicates that the rate of absolute power decline with age in the delta band was similar across IN and NS groups, and throughout all parts of the night. The only study on SO power (<1.25 Hz) found no statistically significant differences between groups[96].
