# Supplementary material for: A Systematic Review and Meta‐Analysis of Biological Sex Differences in Sleep Spindles and Slow Wave Activity in Adults With and Without Insomnia
Source: J Sleep Res. 2026 Jun 22;35(5):e70388. doi: 10.1111/jsr.70388 (PMC13619051; doi:10.1111/jsr.70388)
Supplement: Supplementary file 2 [file JSR-35-e70388-s002.docx]

**Table 1 – Glossary of terms**

| **Biological Sex** | **Physical and physiological attributes** (e.g., chromosomes, hormone levels, reproductive organs, and genitalia) typically categorized as male or female at birth. While most individuals are classified as either male or female, a small percentage are born intersex, meaning they have natural variations in these attributes that do not fit typical biological sex definitions. Biological sex may not align with an individual's gender or gender identity. |
| --- | --- |
| **Gender** | **Societal construct** that encompasses roles, behaviors, expectations, and norms culturally associated with being a man or woman. Varies across cultures and time. |
| **Gender Identity** | **Personal, self-defined, internal sense** **of one’s own gender**, which may or may not align with the sex assigned at birth. It includes identities such as man, woman, both, neither, or somewhere along the gender spectrum. May differ from both their biological sex and societal gender expectations |

*Note. The following definitions are based on established frameworks, including the Canadian Institutes of Health Research (CIHR, 2015) Sex, Gender and Health Research Guide and the American Psychological Association (APA, 2015) Guidelines for Psychological Practice with Transgender and Gender Nonconforming People.*
